# Intersectionality in healthcare leadership: a scoping review on the career experiences of racially and ethnically minoritised women health professionals

**DOI:** 10.1186/s12939-025-02608-x

**Published:** 2025-09-30

**Authors:** Ifeoluwa Adesina, Anju E. Joham, Nada Hamad, Mihirika Surangi De Silva Pincha Baduge, Belinda Garth, Thuy Vy Nguyen, Jacqueline Boyle

**Affiliations:** 1 Department of Health Systems and Equity, Eastern Health Clinical School, Monash University, Level 2, 5 Arnold Street, Box Hill, VIC 3128 Australia; 2 Monash Centre for Health Research and Implementation, Monash University, Melbourne, VIC Australia; 3 Intensive Care, Monash Health, Melbourne, VIC Australia; 4 Department of Haematology, St. Vincent’s Hospital Sydney, Darlinghurst, NSW Australia; 5 St. Vincent’s Clinical School, School of Clinical Medicine, Faculty of Medicine and Health, University of New South Wales, Sydney, NSW Australia; 6 St. Vincent’s Clinical School, School of Medicine, University of Notre Dame Australia, Sydney, NSW Australia

**Keywords:** Women, Leadership, Gender equity, Intersectionality

## Abstract

**Background:**

The underrepresentation of women in positions of leadership, policy, and decision-making is a persistent issue within the healthcare workforce. Disparities in representation are particularly pronounced for women with minoritised racial and ethnic identities. Ensuring the equitable advancement of women into health leadership requires moving beyond approaches that homogenise the experiences of women to addressing the distinct needs of women with intersecting identities. This scoping review sought to summarise the existing evidence on the impact of the intersection of gender and race/ethnicity on the workplace experiences, career journeys, and leadership attainment of women health professionals with minoritised racial and ethnic identities.

**Methods:**

This scoping review was completed using Arksey and O’Malley’s five-stage methodological framework. A comprehensive search identified peer-reviewed papers and grey literature. Inclusion criteria followed an a priori protocol, with no restrictions on publication date, geographic location, or healthcare setting. The search was limited to the English language. A deductive content analysis approach was used to categorise data according to the three forms of intersectionality: structural, political, and representational. Additional categories focused on the psycho-emotional impacts of experiences and women’s agentic responses.

**Results:**

Of the 4043 sources identified, 57 were included in the review. Findings show that racially and ethnically minoritised women in healthcare more frequently described experiences of race-based inequities. This underscores the salience of racialisation in their experiences of marginalisation, an aspect often obscured by gender-only analyses. Current Diversity, Equity, and Inclusion initiatives were reported to have limited effectiveness in addressing the challenges faced by REM women in their careers. These initiatives often lack targeted and impactful strategies to counteract racial and/or gender-based discrimination, provide meaningful support, and promote equitable access to advancement and leadership opportunities. Findings highlighted the prioritisation of Eurocentric/Western knowledge, the prevalence of institutional Whiteness, and practices of tokenistic inclusion. Disproportionate workload allocations, and the burden to consistently outperform White women counterparts, were reported. Persistent exposure to microaggressions, racialised stereotypes, and organisational reluctance to confront racism were also noted. Psychological and emotional challenges, including burnout and internalised oppression, were highlighted. Agency, resilience and resistance were demonstrated through strategic disengagement, professional development, reframing challenges as growth opportunities, forming diversity networks, and advocating for minoritised colleagues.

**Conclusion:**

Advancing gender equity in health leadership requires targeted efforts to address and dismantle racism. Key solutions include integrating antiracism education, recognising non-Western leadership styles, and establishing safe and robust accountability mechanisms. Most evidence in this review reflects the experiences of African American women, underscoring the prevalence of US-centric research. Future studies should investigate other geopolitical contexts.

**Supplementary Information:**

The online version contains supplementary material available at 10.1186/s12939-025-02608-x.

## Introduction

Women form the backbone of the global health sector yet their presence, voices, and expertise remain conspicuously underrepresented in key positions of leadership, policy, and decision-making [1–4]. Despite making up 70% of the global health sector and 90% of health workers in patient-facing roles, women hold only 25% of senior and 5% of top health leadership positions [1, 3, 5]. Disparities in leadership representation are particularly pronounced for racially and ethnically minoritised (REM) women, with progress towards gender equity thus far being of most benefit to White women [6, 7].

In this review, “racially and ethnically minoritised” refers to individuals and communities identifying with any racial or ethnic group other than the historically privileged “White” category [8]. This includes, but is not limited to, Black, Asian, Hispanic/Latinx, Indigenous, and multiracial groups [8]. Beyond a focus on numerical representation, “racially and ethnically minoritised” emphasises the reality that individuals and communities do not naturally exist as minorities, but are actively rendered so by structures and processes that work to systemically erode cultural, economic, political, and social power and identity [9]. Although requiring nuance and further exploration, the term “White” is used here to refer to individuals and communities visibly characterised by European ancestral origins [10]. 

Limited research from the United States and Canada reports that REM women account for 14% of senior managers and 4% of C-suite executive roles in healthcare [11]. In contrast, White women hold 48% and 28% of those roles respectively [11]. Overall, REM women experience the sharpest drop in representation at each successive step in the healthcare career pipeline [11]. 

Numerous factors affect the career progression of women health professionals of all racial and ethnic backgrounds. Enduring patriarchal systems of power perpetuate norms and expectations about the inherent capabilities of men and women, and the types of roles for which they are most suited [12–15]. Organisational policies and practices such as inflexible working arrangements, limited leave entitlements and hiring practices that prioritise men [16, 17], foster gender discrimination, give rise to institutional power imbalances, and restrict women’s agency [12, 16]. Additionally, women face resistance to their leadership, and the devaluation of their credibility and leadership capabilities [16, 17]. Limited encouragement to advance their career, limited access to mentors, sponsors and role models, and limited organisational resources and supports to facilitate their career progression, also impair leadership [12, 14, 18]. Importantly, incidences of sexual and gender-based harassment and assault remain prevalent in healthcare organisations, and women frequently encounter unclear, inaccessible, and inconsistent formal reporting systems [12, 19]. Societal and organisational barriers to women’s career progression can induce low self-esteem and morale [20], hinder leadership identity development [17], and decrease productivity and motivation to pursue leadership [12]. 

Racially and ethnically minoritised women face a disproportionately challenging professional journey [21, 22]. Due to their positionality at the intersection of race, ethnicity, and gender, women from REM backgrounds experience a “double jeopardy” [23], facing both gender - and race - based oppression, perpetuated by the structural forces of patriarchy, colonialism, capitalism, and racism [21, 24–28]. 

Yet, the unique voices, experiences and challenges faced by REM women are often overlooked in discussions surrounding gender equity in health leadership [29, 30], leading to a significant knowledge gap and insufficient efforts to address the barriers faced by these health professionals in their career journeys. Acknowledging and embracing the reality of the intricate interplay of socially constructed identities such as gender, race, and class is essential for developing and implementing effective strategies and initiatives to advance gender equity in healthcare leadership. Failure to do so will maintain the status quo and the likelihood of reinforcing the very inequities that we strive to eradicate.

This review aimed to identify how being situated at the intersection of gender and race/ethnicity impacts the workplace experiences, career journeys, and leadership attainment of REM women health professionals. In doing so, this review will highlight key research, policy, and practice priorities to consider to support the career progression of REM women in healthcare.

## Methods

A scoping review was conducted to identify and summarise existing evidence on how the intersection of race/ethnicity and gender shapes the careers of REM women in healthcare. Scoping reviews provide a systematic approach to mapping the range, nature, and extent of literature in a given field [31], and are recommended for addressing broad research questions, examining emerging or underexplored fields or subject areas, and informing future research directions and policy agendas [31–34]. Preliminary searches revealed a notable absence of comprehensive overviews that explicitly centre the voices and lived experiences of REM women in healthcare as a distinct group. Given the nascent and limited nature of the evidence base on intersectionality within the healthcare context, a scoping review was deemed an appropriate methodological approach. Moreover, this approach supports the identification of knowledge gaps, which directly aligns with this study’s aims to advance knowledge in this area and inform research, practice, and policy.

The protocol for this scoping review was registered through the Open Science Framework (10.17605/OSF.IO/K7JTD*).* The Preferred Reporting Items for Systematic Reviews and Meta-Analysis: Extension for Scoping Reviews (PRISMA-ScR) guided reporting [35]. In accordance with the methodological framework proposed by Arksey and O’Malley [31], and refined by Peters et al., [36] this scoping review followed five stages: (1) identifying the research question, (2) identifying relevant studies, (3) study selection, (4) charting the data, and (5) collating, summarising and reporting the results.

### Stage one: identifying the research question

The following research question guided this review: What evidence exists regarding how gender intersects with racial and ethnic identity to influence workplace experiences, career progression, and leadership attainment of racially and ethnically minoritised women health professionals?

We respectfully acknowledge the complexity and diversity of gender identity, recognising its fluidity and its variation across different societal and cultural contexts. Throughout this paper, the term “woman” is used to be inclusive of all those who self-identify as women. We acknowledge that some individuals assigned female at birth may have a different experience of gender, and conversely, there are those assigned male at birth who identify as women.

### Stage two: identifying relevant studies

An experienced librarian supported the development of the comprehensive search strategy. After identifying relevant articles through an initial limited literature search, keywords and subject headings were extracted to create a full search strategy for MEDLINE (see supplementary file). The search strategy was then tailored for EMBASE, PsycINFO, CINAHL Plus, Sociology database, SCOPUS, and Studies on Women and Gender Abstracts. Hand searches were conducted across the International Journal for Equity in Health, the British Medical Journal, and the Journal of International Women’s Studies. Grey literature sources, such as reports, theses, and dissertations, were also identified via the Social Science Research Network, BASE, OpenGrey, and ProQuest Dissertations &Theses Global. Initial Database and Journal searches were completed in May 2023 and updated in May 2024, using the same search terms and inclusion criteria.

### Stage three: study selection

Following the search, all the identified information sources were collated and uploaded to the Covidence software platform. After removing duplicates, three independent reviewers (IA, MDSDSPB, and JB) screened the titles and abstracts of the remaining sources. Full texts were then assessed (IA, TVN, MDSDSPB, and JB) against the eligibility criteria guided by the Population, Concept, and Context (PCC) framework (Table 1). Conflicts were resolved through discussions until a consensus was reached.

**Table 1:** Eligibility criteria and PCC framework

	Population (*P*)	Concept (C)	Context (C)	Source type
Inclusion	Women healthcare professionals, of any age and professional level, who identify as belonging to a racially and/or ethnically minoritised group.	Sources that provide intersectional data and address any outcomes related to the three forms of intersectionality (structural, political, representational)	The health workforce, with no restriction on geographic location, setting, or organisation type	Primary research sources, no restrictions on methodological approaches No restriction on year of publication
Exclusion	Data from male healthcare professionals Data from non-racially and ethnically minoritised women health professionals Data from traditional and complementary medicine practitioners University students	Gender data not disaggregated by race or ethnicity Race data not disaggregated by gender	Non-healthcare workforce or sector	Non-English language sources without an English translation Conference proceedings and posters Abstracts Literature reviews Commentaries Study protocols Books & book chapters

### Stage four: charting the data

A data charting form was developed in Microsoft Excel by one reviewer (IA) and subsequently pilot-tested by two reviewers (IA and MDSDSPB) using six included sources. The pilot test resulted in a minor update to the charting form. One reviewer (IA) independently charted data from the remaining citations. Data on information source characteristics (e.g. author, publication year, and source type), methodology employed (e.g. study design and methods, if applicable), sample characteristics (e.g. race/ethnicity data and healthcare occupation), and key findings related to the purpose of the review (e.g. intersectional career experiences and implications for research, policy, and practice) were charted. In accordance with the scoping review methodology, a quality assessment of the included information sources was not undertaken.

### Stage five: collating, summarising and reporting results

A deductive content analysis approach was used to categorise the data, with intersectionality serving as the analytical framework.

Coined by Kimberlé Crenshaw, intersectionality provides a critical lens through which the complexity of multidimensional realities can be interrogated and understood [24, 37]. At its core, intersectionality recognises that social identities, such as gender, class, age, race, and sexuality, interact to shape micro-level experiences that reflect and reinforce macro-level systems of oppression and structures of privilege [24, 38]. Existing literature and equity initiatives often analyse gender or race in isolation, obscuring the nuanced experiences of REM women who exist at the nexus of those identities [38]. Applying intersectionality as the analytical framework in this review enables a more comprehensive examination of inequities, bridging critical gaps in current knowledge.

Crenshaw outlines three categories to serve as “analytical guides” [39, p. 3] in the examination of the intricate interplay of social identities: structural, political, and representational [24]. Structural intersectionality relates to the socio-structural systems that engender subordination and disproportionate distributions of privilege among social groups [24, 39–41]. Political intersectionality addresses the invisibilisation of the concerns and agendas of individuals with multiple social identities in unidimensional discourses, policies, and movements centred on justice and equity [24, 40, 41]. Representational intersectionality focuses on the socio-cultural discourses, language, and imagery that reproduce stereotypes which reinforce marginalisation and broader social power relations [24, 39, 41]. 

To ensure accuracy, the first author (IA) engaged in immersion in the data and carefully read all the sources multiple times to gain a comprehensive understanding of their content. Subsequently, key data were identified and mapped according to the three categories.

## Results

**Fig. 1:**
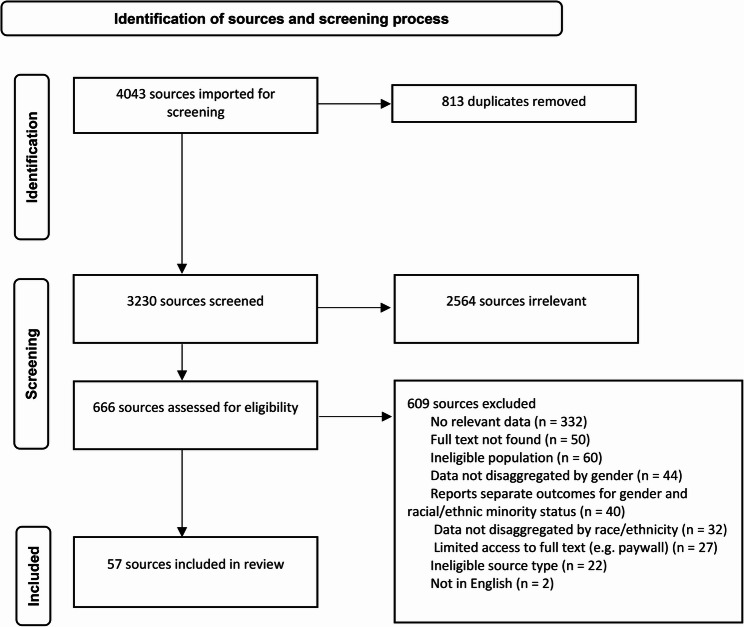
PRISMA flow chart

A total of 57 sources met the eligibility criteria and provided data for this scoping review (Fig. 1). Information sources were published between 1999 and 2024 and originated from the US (*n* = 38), Canada (*n* = 9), the UK (*n* = 8), South Africa (*n* = 1), and Micronesia (*n* = 1). Women of African ancestry were the most represented REM group. Most information sources presented the experiences and perspectives of nurses (*n* = 21), followed by medical professionals (*n* = 20) and those in the allied health field (*n* = 6). The primary research studies included qualitative (*n* = 36) and quantitative (*n* = 12) designs. All 57 sources presented evidence for structural intersectionality, 12 highlighted representational intersectionality, and two addressed political intersectionality. See the supplementary file for summaries of the key findings for each study.

Themes exploring the workplace experiences, career progression, and leadership attainment of women health professionals from REM backgrounds are presented in relation to the three forms of intersectionality: structural intersectionality, political intersectionality, and representational intersectionality. Additional categories focusing on the emotional and psychological impacts of workplace and career experiences, as well as agentic responses to these experiences, are also noted. It should be emphasised that although the three forms of intersectionality are typically treated as distinct concepts, there are some instances in which they overlap.

### Structural intersectionality

*“I think it’s about being a woman*,* and I think it’s about being African American….I had to be better at both. Because both were going to be looked at as second rate.”* [42, p. 6]

All sources provided evidence of structural intersectionality, demonstrating that REM women health professionals experience various forms of oppression rooted in the multiple manifestations of racism. These include (i) ideological racism, (ii) institutional racism, and (iii) interpersonal racism. Although experiences of sexism and gender-based discrimination were acknowledged and often intersected with racism, the women represented in the included sources more frequently reported encountering racial bias and discrimination.

### Ideological racism

*“People look at us differently*,* whether they mean to or not. They look at us as if our ideas aren’t as powerful.”* [43, p. 203]

Beliefs and attitudes that promote and standardise the ideology of the inherent superiority of Eurocentric/Western knowledge systems, values, communication styles, and practices were underlying forces in the professional experiences of REM women health professionals [44–48]. Eurocentric/Western approaches to leadership, which often prioritised individualism, performance, and assertiveness, were perceived to be incongruent with the collectivistic cultural and traditional values such as humble pride, relationality, and community embodied by REM individuals [44, 45, 49]. Some women communicated sentiments of needing to become bicultural tightrope walkers, engaging in a precarious dance of negotiating when and if to express their authentic selves [44, 50, 51]. In the Micronesian context, women nurse leaders working within an increasingly Westernised health care system described the challenge of balancing the often contradictory demands and expectations of Western and traditional values and practices as akin to “learning to walk with their feet in two worlds” [44, p.189] Cultural assimilation to Eurocentric/Westernised ideals was often seen as the only means of survival, with some overcorrecting their behaviours and communication styles to mimic White colleagues in order to gain respect and acceptance [45, 50]. This “whitewashing” [52, p. 92] led to circumstances where the oppressed became the oppressor, as conveyed by a Black woman academic Nurse leader: “I find myself towing the White line and behaving in the same manner that oppressed the Black nurses and students in order not to antagonize them and to save my job” [50, p. 5]. The detrimental effects also impacted self-concept, due to the internalisation of negative racial stereotypes [44, 53]. 

Racially and ethnically minoritised women health professionals faced biased, unjustifiable evaluations of their capabilities and credibility [45–48, 50, 52, 54–62]. This invalidation, explicitly and implicitly communicated by patients, peers, and organisational leadership, was reported to be the result of deeply ingrained prejudices that trivialise the intelligence and contributions of REM individuals [54]. Preconceptions of the value of REM women’s work was also reported to influence the roles to which they were appointed [62, 63]. For example, Prendergast calls attention to the two-tier system within the Canadian nursing workforce, whereby Black nurses are overwhelmingly allocated to underpaid and subordinate positions, while their White counterparts are disproportionately appointed to positions of leadership and management [62]. 

### Institutional racism

Practices, procedures, processes, and both stated and unstated rules and expectations reflecting and reinforcing prevailing racist ideologies at societal levels, were extensively documented [45, 46, 53, 55]. Workplace power relations created insider/outsider dynamics ensuring White healthcare professionals remained central (insiders), whilst individuals with REM identities remained relegated to the periphery (outsiders) [42, 62]. Diversity, Equity and Inclusion (DEI) initiatives implemented within healthcare organisations were perceived as inadequate for dismantling inequitable structures that hindered the leadership advancement of REM women, owing to their failure in addressing entrenched historical biases and cultural complexities [64]. 

#### The invisibility paradox and limited career support

“*I do not really count for much in the grand scheme of things. I am a single spot of color on a very white canvas.”* [65, p. 456]

A common experience shared by REM women health professionals was that of being the only, or one of a few, REM women within their departments or broader organisational leadership structures [46–48, 50, 52, 55, 58, 59, 61, 65–71]. This lack of representation, a form of “environmental microaggression” [72, p. 5], was more prevalent among REM women physicians than their male counterparts [72]. The notable absence of racial and ethnic diversity created a paradoxical experience, leaving REM women health professionals feeling simultaneously hyper-visible and invisible within their organisational contexts; [45, 52, 65] standing out from the predominantly White employee population yet with voices often silenced, presence largely dismissed, and ideas and contributions usually ignored unless repeated by White colleagues [42, 45, 47, 51, 57–61, 65, 66, 73]. 

The underrepresentation of REM women in health leadership restricts the availability of, and access to, mentors and role models who share comparable lived experiences [29, 47, 56, 59, 61, 68, 70, 74, 75]. REM mentors and role models were crucial in advocating for, inspiring, and providing personal and professional support to, REM women at all stages of their career journeys [44, 47, 54, 56, 62, 76]. Additionally, they were reported to help these women navigate organisational power dynamics and the complex interplay of racial and gender-based stereotypes [54, 64]. Bronson’s research focusing on African American women in healthcare administration underscores the connection between mentorship from women with similar racial and ethnic backgrounds, and positive wellbeing outcomes [54]. REM women health professionals viewed the lack of diversity in mentorship as setting them up for failure [66]. Nonetheless, the benefit of “cross-racial mentoring” [44, p. 178] was noted; White mentors provided additional leadership styles and approaches [44], provided allyship and signalled the importance of diversity to other White colleagues [74], and used their privilege to successfully sponsor and facilitate the career progress of REM women in healthcare [59]. 

#### Institutional whiteness and organisational passivity

“*Fighting for visibility in a position that you have already earnt through applications*,* interviews*,* and exams*,* is the cost of being unexpected and not fitting assumptions*” [77, p. 1].

The absence of racial and ethnic diversity at successive levels of leadership hierarchies in healthcare was reported to signify and sustain “institutionalized whiteness” [45, p.199], ultimately mirroring and validating ideological racism embedded within broader societal contexts. A unique situation is observed in South Africa, where the dismantling of apartheid-era policies means that more Black women health professionals are now, for the first time, stepping into roles historically reserved for White individuals [70]. The enduring legacy of apartheid continues to influence the professional trajectories of Black women in South Africa’s health sector, contributing to complex career pathways and delays in reaching leadership positions [70]. Within the Canadian nursing context, persistent hegemonic constructions of the ideal nurse ensured that women who did not adhere to White, middle-class, and heterosexual social identities remained marginalised [62]. Prendergast [62] submits that constructions of the “ideal type” is a “technology within colonial practices” (p.105) that maintains power dynamics, engenders occupational segregation, and ensures the experiences and qualifications of REM individuals are devalued. This institutionalised Whiteness, reflected in healthcare organisations’ reported passivity in dismantling racial power imbalances, fostered a sense of exclusion and cultivated work environments where REM individuals felt hesitant about seeking employment or promotion opportunities [53, 76, 78]. 

#### Inequities in advancement opportunities

Racially and ethnically minoritised women health professionals faced unclear and inequitable promotion processes within their organisations [47, 53, 54, 58, 62, 66, 70, 76, 79–81]. Opportunities for advancement among women nurses, midwives, and pharmacists in the UK and USA were often reported to be largely dependent on personal connections, being part of the “in” group, and being well-liked by those involved in hiring, recruitment, and promotion processes [66, 76, 79, 81]. Despite possessing equal or greater skills and experiences than their White counterparts, REM women health professionals were frequently passed over for promotion [43, 45, 51, 53–55, 58, 66, 73, 79] and denied the respectability afforded to White colleagues [62, 66]. Bakken’s exploration of leadership aspiration among pharmacists in the USA found that regardless of gender, REM pharmacists expressed the highest levels of interest in pursuing leadership yet held the fewest leadership positions [82]. In contrast, White pharmacists were the least interested in leadership but held approximately 80% of leadership roles [82]. 

Research conducted among women health professionals in the USA and Canada indicated that, compared to REM women, White women were more often recommended for leadership [54], more consistently offered leadership positions [80], and were provided with more opportunities to exercise authority and responsibility [47]. REM women in healthcare were seen as confined to “hybrid spaces” – effectively “pseudo-leadership” positions with minimal authority, autonomy, and inclusion in policy and high-level decision-making processes [62]. 

#### Tokenistic inclusion

Institutional racism was further evident in the strategic use of individuals with REM identities to create an illusion of diversity and proffer a “veneer of inclusion” [50, p. 9]. This tokenistic inclusion manifested as: staged photo opportunities intended to portray the organisation as more diverse than was the reality [67], appointments to positions, boards, and committees merely to fulfil targets and quotas [50, 58], appointments to undesirable leadership roles, especially during times of organisational crises [52], leadership roles with little to no support or decision-making authority [52], and the expectation to act as the voice for all REM people [50, 58]. One study highlighted the experiences of Black women nurse leaders who were asked to lead diversity and inclusion committees and initiatives, but were limited in their authority with the condition they only addressed “safe” topics and avoided discussing issues related to race [50]. In some cases, being aware of their tokenisation, REM women in healthcare strategically leveraged this to visually demonstrate the possibility of attaining leadership positions, to amplify the voices and concerns of their communities, and to promote the professional advancement of others from similar backgrounds [50, 67]. 

#### The race burden: internalised expectations and the minority tax

“*I’ve got to get it right because I need to make sure that I’m paving the way for the next person. Sometimes you are the experiment*.” [58, p. 89].

Tokenistic inclusion is closely linked to the concept of the “minority tax”, referring to the disproportionate responsibilities and expectations shouldered by minoritised individuals to uphold diversity within their workplaces [65, 83]. The workload and time demands of these often uncompensated responsibilities and expectations were considered equivalent to the process of earning another degree [46]. Armijo et al. [83] found that among women physicians in the USA, 14.5% women physicians of colour reported being asked to participate in citizenship tasks because of their race, compared to only 0.9% of White women physicians. These tasks often involved voluntary work-related duties that, while advantageous to organisations, could impede individual career advancement by diverting attention from scholarly productivity and other promotion-determining activities [61, 83]. 

While the minority tax is imposed externally, it can be adopted internally. Armijo et al. [83] found that compared to their White counterparts, women of colour physicians were more likely to report feeling obligated to volunteer for citizenship tasks. This internalised sense of obligation stemmed from the intense sense of responsibility to protect, support, serve as role models to, and advocate for, other minoritised individuals [46, 47, 50, 56, 59, 60, 67, 83, 84]. Similarly, REM women health professionals felt a responsibility to speak up for minoritised patients who often expressed frustrations at the lack of cultural competence displayed by White health professionals [62]. Educating White colleagues about racist and discriminatory practices was another example of the additional burden faced by minoritised health professionals [67]. 

REM women health professionals also experienced pressure to be “twice as good and work twice as hard, be prepared twice as much” [60, p.115] than their White counterparts [51, 54, 56, 59, 62, 66, 73, 85, 86]. Iheduru-Anderson and colleagues [50] refer to this phenomenon as the “race burden”. Additionally, these women felt they needed to embody a level of “professional perfectionism” [45, p. 200] not required of their White colleagues, in order to be seen as up to standard, and to receive a modicum of professional recognition [45, 51, 60, 66, 87]. In Bronson’s exploratory study of executive-level African American women in healthcare administration, participants described the pressure to assume a “Black superwoman” or “strong Black woman” persona; juggling emotional constraint and environmental mastery to be seen as just as competent and qualified as their White colleagues [54]. 

### Interpersonal racism

Interpersonal racism refers to the differential treatment, prejudice, and discrimination that individuals encounter due to race or ethnicity during personal interactions. REM women health professionals experience this overtly and covertly, as well as directly and indirectly [46, 55, 71]. The forms of interpersonal racism experienced include (i) othering and social exclusion, (ii) racialised discourses and microaggressions, and (iii) colleague indifference and aversion to addressing racism.

#### Othering and social exclusion

“*They may have allowed us a seat at the table*,* but they will always see us as the others*,* those people.*” [50, p. 6].

The lack of representation of REM women in organisational imagery, work environments, and leadership structures contributed to experiences of being othered [47, 50]. Stewart [66] notes the normativity of exclusionary practices directed at REM health professionals by White colleagues and leaders. The intentional exclusion of REM women health professionals from meetings, decision-making, informal networks, awards and moments of recognition, and social gatherings, was commonplace and sent a message of non-belonging [45–47, 55−57, 59, 66, 85–88]. REM women health professionals were subjected to racial slurs and derogatory language [45, 85], infantalisation [57], sabotage to their promotion [60], and experienced patients refusing care because of their skin colour or accent [45, 54, 60, 68]. 

Internationally qualified women health professionals from low-and middle-income countries face a marked degree of othering and exclusion. They confronted beliefs, policies, and practices implicitly labelling their educational and professional achievements as inferior, consequently delaying their acceptance and integration into both the health workforce and country of migration [47, 57, 62, 89]. 

#### Racialised discourses and microaggressions

“*You are different from those people… I know you are Black*,* but when I talk to you*,* I don’t see you as Black… your thinking is different.*” [46, p. 55].

Microaggressions, defined as “subtle, insulting, discriminatory comments, or actions that communicate a demeaning or hostile message” [72, p. 2], were pervasive in the career experiences of REM women health professionals. These manifested as thinly veiled messages of non-belonging, embedded in seemingly innocuous statements and questions that aimed to interrogate racio-ethnic origins, or educational and professional experiences [46, 75]. Statements such as “you are so articulate” or questions such as “Where are you from?” were frequently encountered by REM individuals, and were reported to be discrediting and marginalising [46, 53, 62, 75]. 

Microinsults, particularly those implying the limited intellectual and professional capacities of REM women, were also well reported [48, 61, 66, 73]. REM women health professionals were frequently assumed to hold lower-level, hospitality, or janitorial jobs within their workplaces and in professional events [48, 54, 57, 58, 61, 66, 68, 86, 90]. Many reported encountering a widespread “cognitive dissonance” [29, p. 573] where their presence in positions of authority contradicted ingrained beliefs and expectations to such a degree that it elicited shock responses from colleagues and patients [29, 58, 68]. Reports of vicarious racism included colleagues’ microinsults associating violence with racialised communities, or characterising racialised patients as poorly educated and primitive [55, 71]. 

#### Colleague indifference and aversion to addressing racism

For REM women health professionals, experiences of interpersonal racism were generally met with opposition, apathy, or scepticism from colleagues and leaders within their workplaces [45, 53, 66]. Organisations were typically slow to respond to reports of racist and discriminatory behaviours, tended to rationalise adverse behaviours, and in some cases showed a direct lack of empathy [45]. REM women health professionals were often admonished for being “too sensitive” or for over-sensationalising their experiences [50, 66]. One participant in Floyd’s study [55] reported that her colleagues expressed being “sick and tired of Black people talking about racism…everybody has their problems” (p. 92). Women who spoke up about their experiences were labelled a problem [46], and some women were instructed to apologise to their White colleagues who took offence when confronted about their discriminatory remarks [50]. Healthcare organisations were reported to prioritise the feelings of White employees to a greater extent than those of employees with REM identities [50, 52]. 

### Sexism and gender-based discrimination

Although not as salient as experiences of racism, sexism and gender-based discrimination were also noted. Culturally prescribed gendered expectations, which required women to remain silent, serve as primary caregivers, and be subservient to men, conflicted with career aspirations [43, 44]. Family responsibilities impacted job choices for women, with some opting for part-time roles and foregoing opportunities for advancement and greater career responsibilities [76]. Within their work environments, power imbalances between men and women were reinforced by the underrepresentation of women in positions of leadership and influence. In the predominantly female nursing workforce, men were observed to progress to leadership faster, and were reported to be better compensated [85]. Hussain’s study among junior doctors in the UK found that men were generally hierarchically superior, and women health professionals were often asked to fulfil secretarial duties for their male colleagues [91]. Additionally, compared to their REM male counterparts, REM women health professionals received poorer treatment [73], were assigned greater workloads [78], experienced more frequent sexualisation [72], and were taken less seriously when they raised complaints about unfair treatment, racism and discrimination [91]. 

### Political intersectionality

Two sources addressed political intersectionality [64, 67]. In DeLany’s oral history study [67], African American women Occupational Therapists reported that while some existing professional networks and coalitions within their organisations afforded them some form of professional validation, they only felt truly accepted and respected when they were “deraced from their biological and cultural heritage”(p. 153). Some of these professional networks failed to acknowledge the contributions of these women, thereby denying them recognition and advancement. In response, these women formed professional networks made up specifically of Black individuals to raise awareness of racism and create safe spaces for discussing topics, experiences, and concerns not usually accepted within mainstream networks. Mathies’ research on Black women in health leadership [64] found that DEI policies and initiatives had limited impact on addressing and mitigating the distinct challenges encountered in their careers. This is primarily due to the symbolic nature of such efforts, their lack of integration into institutional structures and practices, and their failure to acknowledge the historical biases, cultural specificities, and systemic inequities that uniquely influence Black women’s career journeys. The absence of robust accountability mechanisms to ensure that the concept of DEI translates into sustained institutional commitment, rather than a “mere checkbox exercise” [64, p. 105], further undermines effectiveness. To address these limitations, and ensure meaningful and long-term impact of DEI strategies, Mathies underscores the necessity of collective responsibility and the active participation of Black women in the development and implementation of DEI policies and initiatives.

While not directly related to political intersectionality, the importance of creating diversity networks such as those formed by women in DeLany’s study is expressed in the study by Dimitropoulos et al. [68] wherein a participant stated that while colleagues could recognise and empathise with sexism, few understood what it was like to simultaneously experience both sexism and racism from both colleagues and patients.

### Representational intersectionality

*“…it’s a lot sometimes. Just knowing that once you walk in the door with a brown face*,* you’re automatically judged on what you should sound like*,* on what you should look like*,* or how much you should know.”* [54, p. 170].

Fourteen sources revealed that REM women health professionals encounter language and stereotypes that undermine their perceived credibility and suitability for leadership. Black women reported being frequently viewed through the lens of the “angry Black woman” stereotype, which frames assertiveness, self-advocacy, or expressions of dissent as hostile, disruptive, and uncooperative [52, 54, 58, 73]. The confidence of Black women was often branded as arrogance [52, 57, 59], and some women were explicitly directed to “temper [their] enthusiasm” for the comfort of White colleagues [52]. Similarly, Asian American nurses who demonstrated assertiveness were generally mislabelled as argumentative [59]. Stereotypes depicting REM women health professionals as lazy and incompetent were also noted [54, 66, 85, 92]. Typically, the individuality and authority of REM women health professionals were reported to be overshadowed by racial stereotypes [56, 85]. 

As evidenced across multiple sources, stereotypical characterisations of REM women health professionals have significant career implications. These stereotypes not only undermine perceptions of REM women’s professionalism [77], they also constrain their ability to cultivate the social capital necessary for career progression. REM women health professionals reported experiencing isolation within their work environments, with strained collegial relationships limiting access to career support and the formation of professional networks [45, 58]. McAfee [58] notes that stereotypes create opportunities for both conscious and unconscious bias to manifest in promotion processes, leading to the “automatic out-selection”(p.83) of REM women from leadership opportunities. Constructions of REM women as unfit for leadership and teamwork reinforce and legitimise racialised hierarchies within healthcare organisations, contributing to the relegation of REM women into lower-level roles with limited authority influence [54, 62, 63]. 

### Psychological and emotional impacts of experiences

“*Every time you have to confront racism*,* you use up not only a lot of energy at the emotional level*,* you almost have to tap into your limbic system*,* into all that history of rejection and being treated as inferior*,* and deal with the rising anger and hurt and the pain*” [66, p. 137].

REM women health professionals faced significant psychological and emotional challenges due to ideological, institutional, and interpersonal racism. These included emotional exhaustion, chronic stress, and burnout [29, 46, 47, 50, 72, 90]. They also experienced resentment and anger from persistent dismissal and requests to shoulder the burden of diversity [47, 65]. Additionally, these women faced decreased self-confidence and the internalisation of oppression [46, 65, 75, 92], along with hypervigilance against unwarranted scrutiny [46, 52]. The cumulative impact of microaggressions led to fear, distrust and anger [55, 75]. 

A prevailing finding was the loneliness and “profound sense of isolation” [68, p. e917] that REM women health professionals experienced throughout their careers, primarily due to their underrepresentation in educational and professional contexts [43, 47, 50, 52, 54, 61, 65, 68, 71, 75]. Those in leadership positions felt this isolation more acutely, with limited opportunities for peer support [47, 52, 54]. The lack of racial and ethnic diversity in leadership exacerbated imposter syndrome and self-doubt, as REM women health professionals questioned their own competence, given the continued pattern of preferential treatment toward White colleagues [75]. 

### Agency, resilience and resistance

Despite numerous obstacles in their professional careers, REM women health professionals demonstrated resilience. Driven by a sense of purpose and the hope of better experiences for future REM health professionals [43], some chose to focus on factors within their immediate control, such as acquiring further education and professional development to enhance their credibility and ensure that they are well equipped as leaders, mentors, and role models [45, 47, 54, 87]. Some drew strength from traditional and religious practices [44, 58, 66], while others intentionally cultivated assertiveness and self-confidence [44, 59]. In the Micronesian context, despite sustained pressure from White leaders to abandon traditional customs in favour of American norms, women nurse leaders demonstrated resilience by developing innovative leadership approaches that blended both Western and Indigenous values [44]. This enabled them to fulfil their professional responsibilities while preserving their integrity and credibility within their cultural communities [44]. 

Ignoring racist remarks and behaviours, and strategically disengaging from invalidating individuals, environments, and situations, were reported as crucial for survival [45, 46, 93]. REM women health professionals sought and created supportive communities and “brave spaces” [68] with other minoritised individuals [45, 46, 58], allowing them to decompress and share strategies to navigate oppressive and discriminatory work environments.

Internationally Educated Nurses (IENs) of colour created spaces of resistance by conforming to the dominant White culture, often distancing themselves from their foreign qualifications and accents to facilitate full integration into their work environments [62]. This strategic conformity ensured that they retained their positions, allowing them to provide support to other IENs [62]. Reframing adverse experiences as opportunities for growth and role-modelling was also display of resistance [45, 47], as was rejecting negative stereotypes; turning the “angry Black woman” into the “strong Black woman”, and “token” into “the one and only” [58]. 

Despite potential risks to their jobs, some challenged inequitable practices and advocated for themselves and other REM colleagues, students, and patients [47]. They strategically chose when to fight unfair treatment and when to remain silent [46, 56, 92], as described by Gooden, “Learning to pick and choose your battles becomes an art in itself.” [92].

## Discussion

This review draws evidence from 57 sources to examine how being situated at the intersection of gender and race/ethnicity affects the workplace experiences, career progression, and leadership attainment of REM women health professionals. Intersectionality was employed as the analytical framework, as it provides a more nuanced and comprehensive understanding of how race/ethnicity and gender shape the realities of REM women. By moving beyond single-axis analyses that consider gender or race in isolation, intersectionality illuminates the distinct challenges and privileges that REM women may experience, thus supporting the development of more targeted, equitable, and inclusive strategies to advance their careers. Key barriers to leadership were identified, framed by Crenshaw’s three forms of intersectionality: structural, political, and representational [39], as well as the psychological and emotional impacts of adverse workplace experiences. Importantly, this review identified the strengths and agency of REM women health professionals and their resistance to discriminatory practices. Notably, the findings of this review revealed that despite embodying multiple intersecting social and professional identities, race-based inequities emerged more frequently in the accounts of REM women health professionals. The complexity of disentangling intertwined social identities [59], and the difficulty in distinguishing which experiences are attributable to which social identities could account for this [90]. However, it could also be that an individual’s racial identity eclipses all other aspects of their personhood. The challenges faced by REM women health professionals often mirror those encountered by women more broadly - feeling invisible in professional spaces [16, 94–96], facing heightened and unfair scrutiny [17], needing to work harder to prove competence and credibility [16, 17, 95, 97], and navigating biased promotion processes [1, 94, 98]. However, for REM women, these challenges are compounded by and primarily viewed through the lens of their race, leading to more complex forms of oppression. Overall, the findings suggest that greater attention must be paid to addressing racism within broader discussions of sexism and gender equity.

Racially and ethnically minoritised women health professionals experience multiple forms of racism in their work environment. Central to this is ideological racism, which fosters racial hierarchisation, racial antipathy and notions of the racialised “other” [99–101]. Literature from Western and non-Western sociocultural contexts emphasises the foundational role of the ideology of White supremacy in shaping healthcare professions and organisations that centre Eurocentric/Western practices and promote White normativity [102–106]. In the global health sector, conformity to Whiteness is often imposed [107], perpetuating racial hierarchies and power asymmetries [108], and sustaining the concept of the White leader prototype [109]. Ferguson & Dougherty [110] identify Whiteness as the implicit standard, shaping what is considered to be acceptable behaviour and communication in work environments. Cerdeña et al. [111] describe the operationalisation of professionalism as a tool to “police the boundaries of belonging”^(p.575)^. Across different sectors, REM professionals often report the problematisation of their racial/ethnic identities and cultural expressions [110]. Consequently, these professionals are often compelled to embody Whiteness and consistently negotiate unattainable expectations of being White whilst inhabiting a non-White body [110]. As evidenced in this review, to survive and advance in their careers and organisations, REM women health professionals often suppress their authentic selves to conform to Eurocentric/Western norms. Moreover, their intellectual capabilities, professional competence, and authority are frequently undermined due to their skin colour, community of belonging, migration status, and accent.

Ideological racism is intricately woven into institutional frameworks, policies, practices, and procedures, that inconspicuously, and at times overtly, privilege members of the majority of White racial groups. While promoting neutrality, equity, and inclusivity, these frameworks remain governed by racialised norms that perpetuate exclusion and maintain power differentials. REM women health professionals encounter institutional racism in recruitment, retention, and promotion processes, and are often overlooked for promotion and placed in pseudo-leadership roles. Research from the USA shows that institutional racism, reinforced by historical injustices, dictates the career trajectories of professionals with minoritised racial and ethnic identities [63]. Regardless of gender, and across various sectors, REM individuals remain occupationally segregated into lower-paying, menial, and hazardous jobs [63, 112, 113], perpetuating wage inequities [114], limiting leadership diversity, and creating a significant disparity in the availability of REM mentors and role models.

Interpersonal dynamics in healthcare environments often marginalise REM women health professionals, silencing their voices and rendering them invisible. Organisational cultures that prioritise White perspectives exclude REM women from decision-making, recognition, and networks essential for career advancement. Racially charged language and microaggressions from White colleagues, along with stereotypes depicting REM women as aggressive, lazy, and undereducated, contribute to an exclusionary workplace environment and limits professional opportunities and advancement.

Needham et al. [115] present a conceptual model highlighting the cyclical and interactive relationship between ideological, institutional, and interpersonal racism. Ideological racism underpins and justifies both institutional and interpersonal racism, producing race-based inequities through psychological (e.g. chronic stress and diminished self-esteem) and material pathways (e.g. restricted access to resources). These inequities reinforce ideological racism and sustain the cycle of oppression.

To foster equitable work environments that lead to improved workplace experiences and leadership attainment outcomes for REM women health professionals, initiatives adopted by healthcare organisations need a multifaceted approach. This includes recognising significant non-Western leadership approaches, implementing targeted recruitment and promotion strategies, establishing formal mentoring and sponsorship programs, and creating safe spaces and networking opportunities for dialogue and support. Importantly, the onus for change must not rest solely on individual women. While individual-level initiatives that aim to enhance leadership capacity and capabilities (i.e. leadership development programs and mentorship) may be beneficial, there is a need to go a step further to address the systems and organisational levels. Healthcare organisations should firmly commit to dismantling and mitigating racism through robust accountability and reporting mechanisms. Health systems do not operate in isolation from historical, social, and political influences, and the power structures that these influences generate and reinforce [28]. Achieving lasting change requires addressing racism at its ideological foundation through decolonising health systems and incorporating antiracism theory and praxis in healthcare curricula.

Gender inequities in health leadership have significant implications, including the loss of intellectual capital [116], reduced innovation and organisational effectiveness [117, 118], limited health policies and initiatives that address the concerns of women, children, and marginalised groups [119], and delayed realisation of the Sustainable Development Goals (SDG) [2] As globalisation leads to an increasingly diverse patient population, health organisations must include diverse perspectives in leadership to ensure that they address the needs and priorities of the communities they serve, enhance the provision of culturally safe and competent care, improve patient access to healthcare, outcomes and satisfaction, and promote the advancement of new knowledge.

This review was limited by the geographical context and language of the sources. Most sources originated from the USA, Canada, and the UK, and were written in English. The included information sources from Micronesia and South Africa provide valuable insights into contexts where Black and Indigenous populations constitute the demographic majority, yet contend with the impacts of globalisation and the increasing dominance of Eurocentric/Western sociocultural and professional norms. The experiences of REM women in these settings underscore how colonial legacies and global power structures shape leadership pathways and workplace dynamics. These perspectives can offer important insights for understanding other post-colonial and culturally hybrid contexts. Recognising that intersectional experiences are context-specific, future studies should consider exploring the experiences of REM women across a broader range of geographical and socio-political settings. Additionally, further research is needed on the diverse experiences of REM women, particularly those of the First Nations or Indigenous women. Most sources involved Black or African American women and did not account for intra-group differences, obscuring the unique experiences of different racial and ethnic groups. Further research is also needed to understand the influence of additional social and political identities (i.e. disability, class, citizenship status, and religion).

It is notable that two out of fifty-seven included information sources addressed political intersectionality. This signals there is still more work to be done to shed light on the inadequacies of current actions, discussions, and agendas aimed at improving diversity, equity and inclusion in health leadership. Additionally, none of the studies explicitly employed intersectionality as a framework to guide the research process.

In line with the nature of scoping reviews, this review utilised rigorous and transparent methods to map the available evidence. The search strategy, developed with the guidance of an experienced librarian, encompassed seven bibliographic databases, manual searches of three relevant journals, and the identification of grey literature from four repositories. Additionally, three independent researchers reviewed the retrieved sources, and the data extraction form was pilot-tested prior to implementation.

## Conclusion

Historically, efforts to advance gender equity, particularly within the healthcare sector, have largely neglected the perspectives, distinct needs, and challenges faced by women with minoritised racial and ethnic identities. The findings of this scoping review emphasise several key realities: (1) women are not a homogenous group and the factors that impact their workplace experiences and career outcomes cannot be attributed to the influence of their gender alone; (2) Diversity, Equity, and Inclusion (DEI) agendas and initiatives will remain inadequate without the intentional consideration of the nuanced and multidimensional nature of lived experience, and a departure from the implementation of one-size-fits-all approaches; (3) beyond efforts to “fix” the individual woman (i.e. through mentorship and professional/leadership development programs), advancing gender equity in health leadership requires the acknowledgement of historical legacies of oppression, and their influence on current organisational practices, procedures, and processes; and (4) the entrenchment of racism within health systems and organisations cannot be ignored. Facilitating the equitable recruitment, retention, and promotion of racially and ethnically minoritised women health professionals requires the decolonisation of health systems. This can be achieved through the elimination of the ideology of White supremacy, the legitimisation of non-Western leadership approaches, the incorporation of antiracism education, and the implementation of safe, consistent, and robust reporting and accountability mechanisms.

This scoping review has informed a larger study that seeks to holistically explore the individual, interpersonal, organisational, and societal influences on the career experiences and advancement of racially and ethnically minoritised women doctors within the Australian healthcare sector. No sources from Australia were identified in this review. Intersectional experiences are context-specific and are shaped by historical, cultural, and socio-political influences. As such, research in this space must move beyond the predominant US-focused lens. This larger Australian-based study will inform the development and delivery of tailored and context-specific individual and organisational interventions to facilitate and equitably sustain the active participation of racially and ethnically minoritised women within leadership structures in the Australian healthcare sector.

## Supplementary Information

Supplementary material 1.

## Data Availability

The authors confirm that the data supporting the findings of this review are available in this published article and its supplementary information files.
