# Supplementary material for: Intersectionality in healthcare leadership: a scoping review on the career experiences of racially and ethnically minoritised women health professionals
Source: Int J Equity Health. 2025 Sep 30;24:245. doi: 10.1186/s12939-025-02608-x (PMC12487624; doi:10.1186/s12939-025-02608-x)
Supplement: Supplementary file 1 — Supplementary material 1. [file 12939_2025_2608_MOESM1_ESM.zip › MEDLINE Search Strategy_Intersectionality in Healthcare Leadership Scoping Review.docx]

**OVID MEDLINE SEARCH STRATEGY.**

| **Concept 1:** Racially and ethnically minoritised | **Concept 2:** Female | **Concept 3:** Medical professional | **Concept 4:** Intersectionality | **Concept 5:** leadership |
| --- | --- | --- | --- | --- |
| - Racial*.ti,ab - Ethnic*ti,ab - Race.ti,ab - Racis*.ti,ab - Minorit*.ti,ab - (cultur* and linguistic* divers*).ti,ab. - (CALD or BAME or BME or BIPOC or BPOC).ti,ab. - Exp Racial Groups/ - Exp Ethnicity/ - "Ethnic and Racial Minorities"/ - Minority Groups/ - Social identification/ - Cultural diversity/ - Race Factors/ - Exp Racism/ | - Wom?n*.ti,ab - Female*.ti,ab - Gender*.ti,ab - Femini*.ti,ab - Female/ - Women/ - Dentists, women - Physicians, women - Women, working/ - Gender identity/ - Femininity/ - Gender role/ - Women's Rights/ - Gender equity/ - Sexism/ - Sex Factors/ - Feminism/ | - Doctor*.ti,ab - Medic*.ti,ab - Physician*.ti,ab - Clinic*.ti,ab - Nurs*.ti,ab - "allied health".ti,ab - "health* professional*".ti,ab - "health* work*”.ti,ab. - Exp health occupations/ - Exp Health Personnel/ - "Attitude of Health Personnel"/ - Societies, dental/ - Societies, hospital/ - Societies, medical/ - Societies, nursing/ - Societies, pharmaceutical/ | - Intersect*.ti,ab - "critical race theory".ti,ab - CRT.ti,ab. - "feminist theor*".ti,ab. - Exp sociology/ - Exp social theory/ | - Leader*.ti,ab - "Career experience*”.ti,ab - "career progress*".ti,ab - "career advancement".ti,ab - “Training opportunit*”.ti,ab - "career mobility".ti,ab - "career success*".ti,ab - "career ladder*".ti,ab. - Leadership/ - Career mobility/ - "glass ceiling".ti,ab. |
