# Supplementary material for: Intersectionality in healthcare leadership: a scoping review on the career experiences of racially and ethnically minoritised women health professionals
Source: Int J Equity Health. 2025 Sep 30;24:245. doi: 10.1186/s12939-025-02608-x (PMC12487624; doi:10.1186/s12939-025-02608-x)
Supplement: Supplementary file 1 — Supplementary material 1. [file 12939_2025_2608_MOESM1_ESM.zip › Summary of Included Sources_Intersectionality in Healthcare Leadership Scoping Review.docx]

**Table S1: Summary of included research studies.**

| **First author (year of publication)** | **Title** | **Geographic Location** | **Research Aim** | **Methodology/Design** | **Description of Sample**  ***minoritised women*** | **Healthcare occupations represented** | **Relevant findings** | **Form(s) of intersectionality** |
| --- | --- | --- | --- | --- | --- | --- | --- | --- |
| Allen (2003) | The lived experience of Micronesian nurse leaders. | Micronesia | To examine the lived experiences of Micronesian nursing leaders | Qualitative hermeneutic phenomenological field study. Interviews + field notes. *DISSERTATION | 24 Micronesian women nurse leaders | Nursing | ***Gender-based discrimination:*** Women’s roles primarily limited to the home and family. Women expected to remain silent in public, especially in the presence of male leaders. Men held more formal power.  ***Sociocultural expectations:*** Decision/desire to become a nurse fuelled by social, religious, and cultural expectations to care for ill or older relatives and community members.  ***Cultural tensions:*** navigating incongruence between Western and traditional values and leadership styles.  ***Class and socioeconomic influences:*** Lack of financial resources to support nursing education limited access for some participants. Families with higher social status were more likely to have access to education and to have political networks that could promote their interests.  ***Positive supports:*** encouragement and role modelling from figures within immediate and extended family. Caregiving and financial support from husbands and extended family. Sponsors and mentors important for career advancement and development.  ***Psychological and emotional effects:*** acculturative stress, isolation. | Structural |
| American College of Healthcare Executives (2002) | A Race/Ethnic Comparison of Career Attainments in Healthcare Management | USA | To determine if the race/ethnic gap in healthcare management careers has narrowed since 1997 based on a similar group of respondents | Quantitative.  Cross-sectional Questionnaire | 275 Black, 79 Hispanic, 49 Asian, 27 Native American | Health Administration/Management | ***Race/ethnic disparities in income and job satisfaction:*** REM women earned significantly lower salaries than their White counterparts, and reported lower satisfaction with pay, benefits and security. Income gap between White women and REM women widened over time.  ***Racial/ethnic discrimination:*** Nearly half of Black women and a third of Black men reported being denied promotions due to their race/ethnicity, compared to less than 4 percent of White individuals.  ***Sense of belonging and job commitment:*** Black women reported a lower sense of belonging and job commitment compared to Black men, White women, and other REM women. Poorer treatment received after mistake made, and low respect received from supervisors and subordinates.  ***Mentorship:*** Barriers to mentorship varied by race and ethnicity. White men were most commonly cited, by REM women, as their most influential mentor.  ***Gendered barriers:*** Compared to male counterparts, childbearing interrupted women’s careers and restricted their ability to regain status within their organisation, and family obligations hindered openness to accept more responsibility at work.  ***Limited organisational support:*** less support from supervisors and harsher performance evaluations compared to White colleagues. Lack of inter-racial/ethnic collegiality among managers, with REM leaders reporting that White managers do not share vital growth and career-related information. Despite affirmative action policies, Black women observed little improvement in preferential hiring or promotion, while Black men noted some positive changes. | Structural |
| Armijo (2020) | Citizenship Tasks and Women Physicians: Additional Woman Tax in Academic Medicine?. | USA | To evaluate differences in reported citizenship tasks among women physicians due to personal or demographic factors and time spent performing those tasks for work | Quantitative.  Survey. | 17 Asian or Pacific Islander; 51 South Asian or Indian American; 15 East Asian or Asian American; 19 Black or Afro-Caribbean or African American; 9 Middle Eastern or Arab American; 25 Latino or Hispanic American. | Medicine | ***Minority tax:*** 14.5% WOC reported being asked to participate in citizenship tasks because of their race, compared to 0.9% WW. 12.5% WOC perceived their race as a factor in feeling obligated to volunteer for work-related citizenship tasks, compared to 3.4% WW. | Structural |
| Bakken (2023) | Leadership aspiration: An intersectional analysis of racial and gender equity in pharmacy | USA | To apply the intersectionality framework to data collected from the 2019 National Pharmacist Workforce Study (NPWS) to understand the association of gender and race with leadership aspiration among pharmacists, including differences in perceived barriers and attractors for pursuing leadership. | Quantitative.  Survey. | Number of REM women respondents not specified | Allied Health | ***Interest in leadership:*** despite showing the highest levels of interest in pursuing leadership within the pharmacy profession, racially and ethnically minoritised men and women held the fewest administrative positions in pharmacy. White men and women represented the lowest proportion of respondents interested in leadership, yet held over 80% of administrative positions. There is a positive correlation between high student debt and interest in pursuing leadership.  ***Barriers to leadership:*** for all racial and gender groups, except Asian men and women, and Latinos, the most frequently selected barrier to pursuing leadership was the potential conflict with family and lifestyle. The stress associated with leadership roles, and not feeling prepared for such roles, was the most frequently selected barrier for Asian women. | Structural |
| Beagan (2023) | Interpersonal, institutional, and structural racism in Canadian nursing: A culture of silence | Canada | To explore how interpersonal, institutional, and structural racism intersect in the professional experiences of racialized nurses in Canada, and how nurses respond | Critical Phenomenology.  Qualitative.  Interviews. | 13 racialised nurses (12 women, 1 man). Only data explicitly from women was extracted | Nursing | ***Race/ethnicity-based discrimination:*** lack of racial diversity in the workplace or leadership structures contributes to the perpetuation of institutionalised whiteness. Racist slurs, comments and behaviours by patients, colleagues and managers. Pervasive negative assumptions about credibility and capability. Social exclusion. Organisational inaction in addressing racist incidents. Inequitable distribution of workload.  ***Invisibility paradox:*** feeling both hyper-visible and invisible.  ***The race burden:*** need to work twice as hard as White colleagues. Meritocracy as cornerstone of racism.  ***Responses to experiences:*** rise above it, keep head down and don’t speak up. Pressure to counteract stereotypes. Fear of being seen as the aggressor for speaking out about racism; "angry Black women". Assimilation with Eurocentric cultural ideals. Accent modification to manage stigma. Further education to increase professional credibility and authority. Finding and creating community with other racialised individuals. | Structural  Representational |
| Beagan (2022) | Systemic Racism in Canadian Occupational Therapy: A Qualitative Study with Therapists | Canada | To examine experiences of racism in occupational therapy, including coping strategies and resistance. | Critical theory. Qualitative. Interviews | 10 racialised women nurses (3 East Asian; 1 Southeast Asian; 2 African Canadian; 1 Black; 2 West Asian; 1 Latin American) | Allied Health | ***Race/ethnicity-based discrimination:*** Eurocentric ways of doing seen as superior. Professional credibility and authority undermined. Racial disparities in representation. Overt and covert racism by clients, students, colleagues and leaders. Veiled racism in comments about ethnic origins. Organisational inaction towards, and dismissal of, racism. Social exclusion by colleagues. Patient refusal of care due to skin colour and accent. Vicarious racism; discriminatory comments directed at patients and other racialised colleagues.  ***Tokenism:*** exoticism, a form of othering. Minority exceptionalism.  ***Minority tax:*** extra work undertaken to protect other racialised students and staff, and to confront racism within organisation.  ***Psychological and emotional effects:*** hypervigilance, loss of confidence, occupational burnout, frustration, anger, "moral fatigue".  ***Responses to experiences:*** individual and collective self-care activities including yoga, meditation, substance use, art & poetry. Making sense of racism; questioning whether experiences are truly attributable to racism or not, self-blame. Choose battles; politely disengage or actively resist. Joining community of racialised therapists to decompress and share experiences. | Structural |
| Bronson (2020) | An exploratory study of perceptions of eudemonic well-being: A study of African American women in the healthcare administration profession. | USA | To examine how executive-level African American women in the healthcare administration field describe and understand their perception of well-being. | Basic Interpretive.  Qualitative.  Interviews. *DISSERTATION | 8 African American women | Health Administration/Management | ***Race/ethnicity-based discrimination:*** patient refusal of care based on race. Authority, competence and intelligence challenged despite significant experience. Held to a higher standard (for promotion and also day to day). Respect not given. Censored and expected to keep silent. Lack of decision-making power. Assumed to be in a lower-level position. Lack of autonomy in professional settings. Assertiveness viewed as aggression.  ***Diversity networks and social supports:*** limited opportunities to engage with other African American women in leadership. Connection to other African-American women due to shared experience.  ***Minority tax:*** need to prove self and work twice as hard. Pressure to be a Black superwoman.  ***Stereotypes:*** lazy, angry, incompetent.  ***Psychological and emotional effect:*** isolation. | Structural Representational |
| Canli (2024) | Barriers and Challenges Experienced by Latina Nurse Leaders. | USA | To learn about the barriers and challenges Latina nurse leaders have experienced in their ascension into leadership roles. | Qualitative. Descriptive.  Interviews. | 17 Latina Nurses | Nursing | ***Race/ethnicity-based discrimination:*** competence undermined; correlation with pervasive perception of nursing as a profession for White women. Racialised differences in job opportunities received. Invisibility due to lack of representation.  ***Minority tax:*** needing to far exceed the requirements of a particular role, and the expectations of colleagues, for leadership potential to be seen. Accumulation of credentials and higher education degrees were identified as crucial to job mobility.  ***Age discrimination:*** Being young was identified as a barrier, unspoken assumption that younger age correlates to low leadership capability.  ***Motherhood:*** caring responsibilities, especially as young or single mothers, were perceived to have a limiting impact on career trajectory.  ***Lack of mentorship:*** underrepresentation of Latinx people in leadership leads to low availability of mentors with similar life experiences.  ***Psychological and emotional effect:*** imposter syndrome; being the only REM person in work settings or leadership engenders low confidence and self-doubt in their capabilities. | Structural |
| Collins (2004) | Career mobility among immigrant registered nurses in Canada: Experiences of Caribbean women. | Canada | To investigate the experiences of immigrant women from the Caribbean who are registered nurses (RNs) in Canada. | Qualitative. Interviews. *THESIS | 14 Caribbean women nurses | Nursing | ***Disparities in representation:*** underrepresentation in organisational hierarchies and imagery.  ***Race/ethnicity-based discrimination:*** White colleagues given more opportunities to enact leadership. Exclusion from opportunities that facilitate mobility (lateral and upward). Legitimacy of professional identity and status often questioned by patients. Voices silenced in decision making processes. Devaluation of knowledge and skills. Foreign-trained nurses seen as "other".  ***Limited organisational support:*** not supported or encouraged to apply for leadership. limited availability of mentors, role models, and support systems of women of colour.  ***Tokenism and minority tax:*** the few in leadership perceive themselves to be tokens, are overscrutinised, and feel a constant need to prove themselves. *Psychological and emotional effects:* anger, frustration, thoughts of leaving the profession.  ***Responses to experiences:*** Coping strategies, including acquiring further education so that they are better equipped for their roles, and potential leadership opportunities. Challenging exclusionary behaviours, decisions and norms. Pioneering pathways to career advancement. Remaining silent so as not to “rock the boat”. | Structural |
| Crown (2021) | The Role of Race and Gender in the Career Experiences of Black/African American Academic Surgeons: A Survey of the Society of Black Academic Surgeons and a Call to Action. | USA | To determine the role of race and gender in the career experience of Black/African American academic surgeons and to quantify the prevalence of experience with racial and gender bias stratified by gender | Quantitative.  Cross sectional survey. | 31 Black/African American women, 22 Black/African American men | Medicine | ***Race/ethnicity-based discrimination:*** racial bias experienced by both Black/African American men and women surgeons however, gender bias was more prevalent among the women.  ***Disparities in career trajectory:*** Black/AA male surgeons were more successful in achieving full professorship compared to Black/AA women surgeons.  ***Career development:*** Participation rates in career development programs and mentorship were similar for both Black/African American men and women however, more Black women doctors expressed uncertainty about the necessary steps to reach their career goals. | Structural |
| DeLany (1999) | African-American women in a predominantly Caucasian female profession: learning paths to positions of prominence. | USA | To understand the learning paths of African American women who held positions of prominence within a predominantly Caucasian, female profession. | Qualitative Phenomenologically based oral history.  Interviews. *THESIS | 22 African American women | Allied Health | ***Disparities in representation:*** the only racial minority/racial minority woman in positions of influence.  ***Race/ethnicity-based discrimination:*** microaggressions. Low expectations of capability. Sexism occurrences were less frequent (compared to racism) but still present in the broader healthcare sector.  ***Minority tax:*** perceived responsibility to mentor and create support systems for other Black students and practitioners. Sense of responsibility to serve others ingrained by parents and primary caregivers. Extra task of educating White colleagues about racism.  ***Tokenism:*** being put forward to be "the face" of diversity for the organisation.  ***Diversity networks and social supports:*** needs and experiences not considered by White networks and professional coalitions. | Structural Political |
| Dill (2022) | Structural Racism And Black Women's Employment In The US Health Care Sector. | USA | To describe how structural racism and sexism shape the employment trajectories of Black women in the US health care system. | Quantitative Analysis of survey data from the American Community Survey | 125,800 men and women working in the health sector (total number of Black women not specified) | Not specified (healthcare in general) | ***Occupational segregation:*** healthcare in the USA is a key employer of Black women. Black women are concentrated in lower-level, lower-paying and higher-risk positions compared to other women and all men from different racial backgrounds. Links to the legacy of slavery in the United States. | Structural Representational |
| Dimitropoulos (2022) | Equity, diversity and inclusion of pediatric clinician-scientists in Canada: a thematic analysis. | Canada | To define the unique opportunities and challenges of pediatric clinician–scientists related to equity, diversity and inclusion; and to identify key components of training needed to support people from equity-seeking groups as emerging and early-career pediatric clinician–scientists to generate diverse health research leaders in knowledge generation, implementation and translation. | Qualitative descriptive. Interviews | 39 paediatric clinician-scientists. Relevant data from the experiences of 5 racialised women (Black, East Asian, Asian, and Spanish) | Medicine | ***Disparities in representation:*** underrepresentation. Limited availability of role models and mentors with shared experiences.  ***Race/ethnicity-based discrimination:*** assumed to hold lower-level jobs. Patient refusal of care due to accent or skin colour. Work dismissed and undervalued.  ***Psychological and emotional effects:*** isolation. | Structural |
| Dolinta (2024) | The Journey and Experiences of Female Hispanic Nurse Leaders. | USA | To understand the experience of female Hispanic nurses in formal leadership roles. | Qualitative.  hermeneutic phenomenology | 15 Hispanic women nurses | Nursing | ***Cultural conflicts:*** Women struggled to conform to culturally prescribed gender roles and expectations related to being a woman; women were expected to aspire to looking after their families, as opposed to advancing their careers.  ***Race/ethnicity-based discrimination:*** often passed over for career opportunities despite having the qualifications. Opportunities given to less qualified colleagues. Organisational inaction to improve diversity.  ***Family supports:*** parents provided unconditional support despite career aspirations deviating from cultural expectations related to motherhood. Mothers taught attributes related to independence and resilience.  ***Leader/peer supports:*** career journeys, superiors and peers were a source of encouragement, confidence, motivation, and mentorship.  ***Psychological and emotional effects:*** isolation, imposter syndrome, resentment due to organisational inactivity to increase diversity, burden of constantly needing to prove self, hope for the future and for other Hispanic people coming through the career pipeline. Resentment at organisational inaction. | Structural |
| Dombeck (2003) | Work narratives: gender and race in professional personhood. | USA | To investigate nurses’ understanding of their culture and professional experiences in the context of the images of nursing in the society at large | Qualitative Ethnographic study. Interviews + participant observation +focus groups + written narratives | 36 nurses (12 African American women) | Nursing | ***Disparities in representation:*** underrepresentation; being "the only one".  ***Race/ethnicity-based discrimination:*** presumed to be suited to lower-status positions. Underappreciated. Leaders' ignorance and indifference in addressing racism. Devaluation of knowledge, intellect, and abilities. Sexual harassment. | Structural |
| Dunkley (2024) | Outsiders Within: The Lived Experience of Being Black and Female When Becoming a Nurse Executive. | USA | To explore the lived experience of being Black and female when becoming a nurse executive, specifically the nuances of being both a racial and gender minority. | Qualitative. Hermeneutic Phenomenology. | 10 Black women nurses | Nursing | ***Minority tax:*** socialised to maintain a high standard of excellence. Failure to achieve career success seen as a reflection of family and community. Arming self with education, job experience, and relevant skills, to exceed expectations and not confirm racist stereotypes. Overwhelming sense of responsibility towards minoritised patients and colleagues. Pressure to represent minoritised individuals in the best way.  ***Race/ethnicity-based discrimination:*** racial slurs, exclusion from organisational initiatives. Undervalued and disrespected. Overlooked for career opportunities. Employees have higher expectations of productivity and work ethnic from Black individuals, compared to White counterparts.  ***Limited organisational support:*** dissatisfaction with the guidance and resources provided by organisations; not sufficient for career growth. Individuals by themselves sought out opportunities to gain professional experience, expand skill sets, and gain visibility within the organisation.  ***Stereotypes:*** participants felt that their race and gender predisposed them to be stereotypes as inadequate and incompetent. “angry Black woman”.  ***Responses to experiences:*** challenge negative opinions and stereotypes about Black women. “code-switching”, adapting persona to fit in. Internal motivation; positive self-perception, self-confidence, spirituality.  ***Psychological and emotional effects:*** Situational awareness; constant consciousness of identities. Not being able to express authentic self. | Structural  Representational |
| Feld (2023) | Parental leave, childcare policies, and workplace bias for hepatology professionals: A national survey. | USA | To understand the influence of parental leave and child-rearing on career advancement in hepatology. | Quantitative.  Cross-sectional survey | 130 women in hepatology (number of REM women not specified) | Medicine | ***Bias:*** Black and Hispanic women more frequently reported receiving unequal pay or benefits, and lack of consideration for promotion or management roles, than their White counterparts.  ***Gender-based discrimination:*** Sex and maternity bias in the workplace and during training interviews were more frequently experienced by Black and Hispanic women. | Structural |
| Floyd (2020) | Empowering Nurses of Minority in the Face of Incivility and Bullying: Through the Lens of Phenomenology | USA | To explore how nurses who represent minorities share their lived experiences of incivility, bullying, and empowerment in the workplace. | Qualitative Descriptive phenomenology.  Interviews. *DISSERTATION | 15 minority women | Nursing | ***Disparities in representation:*** being either the only minority nurse, or one of a very few.  ***Race/ethnicity-based discrimination:*** voices and ideas are unheard. Powerlessness. Lack of professional acknowledgement. Encountering colleague aversion to addressing racism. Microaggressions. Facing negative assumptions about knowledge and competence. Derogatory remarks from White colleagues about minority nurses or patients from minority backgrounds. Perception that organisations condone racism due to inaction.  ***Inequitable promotion processes:*** overlooked for advancement despite having the required skills and qualifications. Often assigned the most challenging tasks and patients.  ***Minority tax:*** Constant need to prove oneself and take on additional work due to being undervalued.  ***Psychological and emotional effects:*** constant stress, sense of defeat. | Structural |
| Fowler (2020) | Facilitators and barriers to leadership and career opportunities in minority nurses in public health departments. | USA | To elicit information from minority public health nurses regarding experiences and opportunities for leadership development and career advancement in public health departments | Qualitative.  Interviews. | 39 minority women nurses (18 African American/Black or Mixed race, 12 Hispanic or Latina, 7 Asian, 2 Indian heritage) | Nursing | ***Race/ethnicity-based discrimination:*** Overt and implicit. Ideas and contributions not acknowledged. Inequitable hiring and promotion processes; despite possessing greater skills and experiences minority nurses are passed over for opportunities. Derogatory remarks.  ***Stereotypes:*** "angry Black woman".  ***Responses to experience:*** Engaging with professional organisations that offered leadership training for minority nurses. | Structural Representational |
| Galloway (2022) | Through the Lens of Servant Leadership: African American Female Nurse Leaders’ Journey to Executive Status | USA | To explore the lived experiences of female African American executive nurse leaders. | Qualitative. Phenomenology. Interviews | 8 African American women | Nursing | ***Limited organisational support:*** limited guidance on how to navigate job requirements and achieve career goals.  ***Minority tax:*** working twice as hard as White colleagues to receive recognition. Authority undermined. Undue performance pressures.  ***Invisibility tax:*** feeling unseen and devalued; ideas only recognised if they come from White colleagues.  ***Responses to experiences:*** Remaining true to authentic self. Establishing relationships and consistency. Maintaining a fearless attitude. Continued efforts to receive credibility; acquiring further education and skills, attending conferences. Informal networks provided solidarity. Faith, family and friends acted as sounding boards. | Structural |
| Gogoi (2024) | Discrimination, disadvantage and disempowerment during COVID-19: a qualitative intrasectional analysis of the lived experiences of an ethnically diverse healthcare workforce in the United Kingdom | UK | To understand how HCWs have been impacted by the pandemic in relation to their social positions by utilising an intersectional approach. To examine how the power dynamics and interaction within and between these social categories (such as ethnicity, occupational role, age and gender) at multiple ecosystemic levels have an influence on HCWs’ experiences | Qualitative.  Interviews, focus groups. | 101 women (number of REM women not specified) | Not specified (healthcare in general) | ***Race/ethnicity-based discrimination:*** reduced agency; not being heard, unable to voice opinions, decisions undermined. Alienation. Overlooked for promotion. Microaggressions. Negative assumptions about capabilities made by patients due to skin colour and origin.  ***Gender-based discrimination:*** Men from racial and ethnic minority backgrounds are perceived to be treated better.  ***Limited social support:*** lack of support with childcare makes it challenging to balance roles with family responsibilities. Misconceptions about ethnic minority support networks; support networks usually overseas.  ***Stereotypes:*** aggressive, angry, unqualified.  ***Psychological and emotional effects:*** lack of sense of belonging. | Structural  Representational |
| Golightly-Jenkins (2003) | An investigation of the relationship among decisions to accept or decline promotion to nursing management or leadership positions and minority women professional nurses. | USA | To investigate differences in offers of promotion and differences in acceptance rates to nursing management or leadership positions among minority and non-minority women professional nurses. | Quantitative.  Survey. *DISSERTATION | 1019 women nurses  166 (16.3%) Black, 128 (12.6%) Other*)  *Other = American Indian/Alaska Native, Native Hawaiian/Pacific Islander, and Mixed/Biracial | Nursing | ***Disparities in promotion:*** White nurses received offers for managerial or leadership positions at a notably higher frequency compared to REM nurses. No statistically significant variations between White and REM nurses in acceptance rates of offers of promotion. | Structural |
| Hassouneh (2014) | The experiences of underrepresented minority faculty in schools of medicine. | USA | To describe and provide evidence for the three major components of our theory of how faculty of color survive and thrive in health professions education, using examples of URM medical school faculty | Qualitative Grounded theory.  Interviews | 16 URM faculty.  9 URM women, all African American | Medicine | ***Race/ethnicity-based discrimination:*** viewed through the lens of skin colour; individuality eclipsed. Negative perceptions about knowledge, competence, and capabilities based on racial group stereotypes. Invalidation of voice and clinical judgement. Unequal treatment and performance standards.  ***Othered:*** excluded from social activities, meetings, and decision making.  ***Diversity networks and social supports:*** networks of non-minority staff are safe spaces where women can discuss experiences of racism and other challenges.  ***Psychological and emotional effects:*** isolation, feelings of loneliness and marginalisation.  ***Responses to experiences:*** strategically and intentionally choosing when to engage and disengage. Maintaining authenticity and staying true to values. Engagement with mentors, professional organisations, colleagues of colour and support networks, were important to thriving within the organisation. Disengagement, including distancing self from discriminatory environments and individuals, is a coping mechanism. | Structural Representational |
| Henry (2007) | Institutionalized disadvantage: older Ghanaian nurses' and midwives' reflections on career progression and stagnation in the NHS. | UK | To explore the perceptions of career progression in the NHS of a group of midwives and nurses trained in Ghana and working in the UK. | Qualitative. Interviews. | 20 Ghanaian nurses/midwives (number of women not specified, only data explicitly from women were captured) | Nursing/midwifery | ***Inequitable promotion processes:*** challenges with being promoted. Lack of transparency in promotion; not based on merit. Cultural differences in communication skills hinder advancement.  ***Limited organisational support:*** inadequate career development training and support, compared to White colleagues. | Structural |
| Hoang (2023) | Exploring the Leadership Paths and Experiences of Underrepresented Minority Physical Therapists. | USA | To explore the pathways of underrepresented minority physical therapists to leadership positions and characterizes the factors influencing leadership development. | Qualitative.  Interviews. | 17 physical therapists (10 women) | Allied Health | ***Race/ethnicity-based discrimination:*** microaggressions, constant comments about physical appearance, patients exhibiting fear/apprehension due to therapists’ physical appearance.  ***Disparities in representation:*** underrepresentation of minority physical therapists; challenges with finding mentors.  ***Minority tax:*** working harder than non-minority counterparts to achieve success. Frustrations about constantly raising awareness of importance of diversity. | Structural |
| Howells (2018) | Exploring the career choices of White and Black, Asian and Minority Ethnic women pharmacists: a qualitative study. | UK | To understand if the career choices of White and BAME women pharmacists are influenced by different factors. | Qualitative. Interviews. | 28 women (3 Black African/Caribbean, 6 Pakistani, 4 Indian, 1 Asian other, 1 Mixed race, 1 Chinese, 2 Middle Eastern) | Allied Health | ***Cultural ideals and career choices:*** values around motherhood and self-employment influenced employment preferences of BAME women pharmacists.  ***Motherhood:*** family responsibilities influence preference for part-time work, and low prioritisation of career progression.  ***Career progression:*** limited evidence that BAME women pharmacists are more likely to experience barriers to career advancement.  ***Gender-based discrimination:*** seen as inevitable, and a consequence of working part-time. Lack of flexibility to support re-entry into the workforce after having children.  ***Inequitable promotion processes:*** sense that career progression is not based on meritocracy. but on "who you know".  ***Race/ethnicity-based discrimination:*** majority of women had not experienced this form of discrimination, but observed inequities in promotion processes, and predominantly White leadership structure, discouraged some from seeking promotion.  ***Importance of diverse leadership:*** having a leader of the same ethnic origin thought to have a positive effect on leadership development and advancement. | Structural |
| Hussain (2023) | The Impact of Race and Gender-Related Discrimination on the Psychological Distress Experienced by Junior Doctors in the UK: A Qualitative Secondary Data Analysis. | UK | To explore the impact of racial or gender-based discrimination on psychological distress experienced in the junior doctor population | Qualitative.  Interviews | 11 women (number of REM women not specified) | Medicine | ***Race/ethnicity-based discrimination:*** microaggressions; peers refusing to attempt to pronounce name.  ***Gender-based discrimination:*** being mistaken for nurses or secretaries, discouragement from entering competitive medical specialties, inappropriate sexual comments from male colleagues, asked to complete secretarial jobs by male leaders  ***Psychological and emotional effects:*** nervousness due to constantly anticipating discrimination, shame, powerlessness, overwhelm, vulnerable, loss in confidence, imposter syndrome, overcompensating by projecting overly tough and hypermasculine behaviours.  ***Responses to experiences:*** less likely to challenge or report poor behaviour due to psychological and emotional impacts, choosing to pick battles. | Structural |
| Iheduru-Anderson (2022a) | The voice of Black academic nurse leaders in the United States: A qualitative study. | USA | To examine the everyday lived experiences of Black academic nurse leaders in the United States through the Critical Race Theory perspectives | Qualitative narrative research.  Interviews. | 34 Black women | Nursing | ***Race/ethnicity-based discrimination:*** authority, credibility and decisions undermined. overscrutinised. Expected to be grateful. Prioritisation of White women's needs (e.g. asked to tone down confidence to not intimidate, asked to apologise when assertive).  ***Invisibility paradox:*** feeling hyper-visible due to disparities in racial representation.  ***Minority tax:*** internalised pressure to be perfect in order to be accepted, and to avoid being a negative representation of all Black people.  ***Tokenism:*** appointed to leadership only in times of crisis or to complete undesirable work. Given responsibilities without appropriate compensation. Not credited for work. Excluded from decision making processes (silenced).  ***Behavioural changes:*** altering actions and communication styles to align with the White majority’s expectations.  ***Stereotype:*** "Angry Black Woman".  ***Psychological and emotional effects:*** hypervigilance, feeling exploited, isolated.  ***Responses to experiences:*** staying silent to avoid retaliation. | Structural  Representational |
| Iheduru-Anderson (2022b) | Diversity and Inclusion or Tokens? A Qualitative Study of Black Women Academic Nurse Leaders in the United States. | USA | To examine how race and gender influence how Black women academic nurse leaders’ function in their leadership positions, how they are perceived by their peers, and how their perception of race, gender, class, and power influences diversity, equity, and inclusion initiatives in the workplace | Qualitative Narrative inquiry.  Interviews. | 34 Black women | Nursing | ***Race/ethnicity-based discrimination:*** overscrutinised, devaluation of authority, reports of racist/discriminatory encounters judged as untrue or an over exaggeration, asked to apologise to White colleagues after confronting them about their discriminatory behaviours.  ***Disparities in representation:*** perpetually othered; being the only/one of a few Black employees.  ***Value incongruence:*** choosing between conforming to White standards or staying true to self. Leadership seen as a form of subjugation.  ***Minority tax:*** expected to provide perspective on the thoughts and behaviors of all people of colour. Expected to take charge of diversity and inclusion initiatives. Utilised to create a facade of diversity without being given the authority to effect real change. Feeling obligated to foster an inclusive environment for themselves and Black peers/students, often with little or no backing from organisation.  ***Limited organisational support:*** limited resources available to Black women, compared to their White counterparts. Lack of White allies.  ***Psychological and emotional effects:*** loneliness, emotional exhaustion, mental health challenges, fear, driven to quit.  **Responses to experiences:** overcorrecting behaviours to be like White colleagues in order to be accepted; not showing vulnerability to not be exploited, fear of repercussions during reappointment and promotion hearings. | Structural |
| Iheduru-Anderson (2023) | Mentoring Experience for Career Advancement: The perspectives of Black Women Academic Nurse Leaders. | USA | To examine how Black women academic nurse leaders perceive mentoring in academic nursing using critical race theory as the guiding framework. To explore the critical role of mentorship in promoting and advancing Black women academic nurse leaders. | Qualitative.  Narrative.  Interviews | 34 Black women | Nursing | ***Race/ethnicity-based discrimination:*** often overlooked and passed over for career opportunities despite having high levels of achievement and intellect.  ***Disparities in representation:*** limited availability of Black mentors is seen as a disadvantage. Lack of representation leads to minoritised nurses needing to act as role models to students and peers.  ***Positive mentoring experiences:*** mentors perceived to be instrumental for career advancement, provide support, guidance, and encouragement, and model appropriate professional behaviours. Mentors also share learning and job opportunities. Black mentors more likely to understand and relate to experiences, fears, and limitations.  ***Positive supports:*** Ethnic professional nursing organisations provided empowerment, as well as opportunities to access mentors and social supports from similar backgrounds.  ***Negative mentoring experiences:*** formalised mentoring structures not perceived to be beneficial. Mentorship from someone with different racial background presents challenges; lack of understanding, undermining, and lack of connection. Individuals left to seek mentorship support by themselves.  ***Psychological and emotional effects:*** discouragement, diminished sense of belonging, race burden; constantly needing to be a positive representation of the Black community.  ***Responses to experiences:*** serving as role models and supports to each other. | Structural |
| Larsen (2007) | Embodiment of discrimination and overseas nurses' career progression. | UK | To examine empirically and in-depth how discriminatory attitudes and practices are experienced by overseas nurses and how the discrimination may affect their well-being and career progression and, furthermore, to apply the theoretical perspective of embodiment in understanding these processes. | Qualitative. Phenomenology Interviews | 2 Nurses (1 male, 1 female. Only data from female extracted) | Nursing | ***Race/ethnicity-based discrimination:*** skills and knowledge not recognised, treated as less qualified despite having the necessary experiences, personal and professional authority consistently undermined, assumed to be in a lower-level job, socially excluded; made invisible, overscrutinised.  ***Psychological and emotional effects:*** internalisation of discrimination, loss of confidence. Hesitance to apply for promotion due to bullying. Remaining silent and not speaking out due to fear of reprimand. | Structural |
| Mathies (2023) | Combating DEI Structures: A Tale of Black Women’s Journeys in Healthcare Leadership | USA | to examine the barriers for Black women to advance to leadership in healthcare as they relate to pipeline practices, engagement, and policies and programs implemented by the industry following the establishment of diversity, equity, and inclusion structures. | Qualitative.  Interviews.  *DISSERTATION | 25 Black women | Not specified (healthcare in general) | ***Race/ethnicity-based discrimination:*** microaggressions, racial pay disparities  ***Limited organisational support:*** lack of mentorship opportunities and sponsorship impacts career opportunities, ability to successfully navigate power dynamics, and availability of psycho-emotional support.  ***Stereotypes:*** historical biases that paint Black women as caretakers, aggressive and confrontational.  ***Ineffective Diversity, Equity and Inclusion initiatives:*** limited success of historical and contemporary DEI efforts in removing barriers and improving career advancement outcomes for Black women. | Structural  Representational  Political |
| McAfee (2021) | Leadership Achievement in Healthcare: An Interpretative Phenomenological Analysis of the Voices and Experiences of Black Women Leaders | USA | To discover the barriers and biases that do not encourage Black women leaders to advance into leadership positions in healthcare organizations. | Qualitative. Phenomenology. Interviews + Artifacts. *DISSERTATION | 12 Black women | Not specified (healthcare in general) | ***Race/ethnicity-based discrimination:*** overlooked for promotion. Expertise questioned.  ***Tokenism:*** perceived to be in role to achieve organisations diversity quota, being the only/one of a few yet expected to represent all Black people. Voice dismissed in meetings.  ***Stereotype:*** being seen as the "Angry Black Woman" hinders career mobility yet its embodiment is perpetuated by the racism embedded within organisations (i.e. racism results in feeling angry and frustrated, speaking out against discrimination).  ***Responses to experiences:*** rejecting stereotypical labels and creating new labels to highlight strength, resilience, empowerment, and sense of pride of being a source of inspiration to other Black women coming through the ranks. | Structural Representational |
| Naresh (2019) | Factors Leading to Successful Asian American Women Leaders. | USA | To identify qualities, experiences, and obstacles that led Asian American women to achieve positions of leadership. | Qualitative phenomenology.  Interviews. *THESIS | 7 Asian American women | Nursing | ***Race/ethnicity-based discrimination:*** devaluation of experience. Credibility and capability questioned. Unequal pay compared to White counterparts. Perpetually othered.  ***Disparities in representation:*** being the only/or one of a very few minority women. Lack of exposure to mentors with similar lived experiences.  ***Minority tax:*** need to mentor, create professional opportunities, and advocate for other Asian American women.  ***Limited organisational support:*** limited access to organisational systems and influential/leadership networks.  ***Psychological and emotional effects:*** isolation, feelings of not belonging. | Structural |
| Nirmul (2023) | Leadership Development of Women of Color in Occupational Therapy: A Qualitative Intersectional Analysis. | USA | To understand which intersecting patterns of social identity categories have shaped the leadership development of women of color leaders in occupational therapy in the United States and how they do so. | Qualitative.  Narrative Interviews. | 8 women (2 African Caribbean, 1 Asian American, 1 Native American, 1 Indigenous White/Hispanic, 2 African American, 1 South Asian) | Allied Health | ***Disparities in representation:*** lack of representation of women of colour in leadership seen as a symbolic representation of non-belonging.  ***Minority tax:*** needing to work harder than White colleagues despite having the same, or greater, amount of qualifications.  ***Positive supports:*** mentors and supervisors were a way to gain social capital, provide career opportunities, and facilitate leadership development. Being seen/recognition of humanity and challenges arising from power relations within society and in organisations.  ***Responses to experiences:*** perseverance; standing up to power relations and navigating microaggressions. | Structural |
| Nunez-Smith (2009) | Health care workplace discrimination and physician turnover. | USA | To examine the association between physician race/ethnicity, workplace discrimination, and physician job turnover. | Quantitative.  Cross sectional survey. | Number of REM women not specified | Medicine | ***Race/ethnicity-based discrimination:*** compared to White women, a higher proportion of Black women reported at least 1 job turnover due to experiences of discrimination. Compared to White women, a higher proportion of Asian, Hispanic/Latina, and Other women reported job turnover due to discrimination. However, this was not statistically significant. | Structural |
| Ode (2022) | Perception of Racial and Intersectional Discrimination in the Workplace Is High Among Black Orthopaedic Surgeons: Results of a Survey of 274 Black Orthopaedic Surgeons in Practice. | USA | To describe the workplace environment and the extent of perceived occupational opportunity and workplace discrimination experienced by Black orthopaedic surgeons in the United States. | Quantitative. Survey. | 58 Black women | Medicine | ***Race/ethnicity-based discrimination:*** devalued, not seen as equal. Black women reported higher rates of microaggressions, compared to Black male surgeons. Microinsults were the most common microaggressions. Assumed to hold a nonphysician or lower-level job. Black women reported that it was difficult to distinguish between their experiences with sexism and racism.  **Limited organisational support:** Black women reported receiving lower occupational opportunity, when compared to Black male surgeons.  **Psychological and emotional effects:** exhaustion due to navigating challenges. | Structural |
| Osborne (2008) | The career development of Black female chief nurse executives | USA | To explore the career paths of Black female chief nurse executives with a view of understanding the factors which both facilitate and hinder the career development of these leaders in healthcare organizations. | Qualitative.  Phenomenology.  Interviews.  *DISSERTATION | 10 Black women | Nursing | ***Race/ethnicity-based discrimination:*** most prominent barrier encountered relates to race. Authority challenged. Capability questioned. Invisible; voice disregarded, invalidation of recommendations. Patients' refusal of care. Feeling othered (us vs you). Gender bias was not generally experienced due to the predominantly female profession.  ***Minority tax:*** Pressure to be twice as good as white colleagues. Need to be overqualified to succeed.  ***Positive supports:*** mentors and role models crucial in career development. Collegial relationships were sources of subjectivity and emotional strength.  ***Responses to experiences:*** coping mechanisms, including reframing barriers as opportunities. Not perceiving self as victim. | Structural |
| Pattani (2022) | Effect of race, gender identity, and their intersection on career satisfaction: A cross-sectional survey of academic physicians. | Canada | To understand how the intersection of self-identified gender identity and race/ethnicity affects career experiences of physician members of the largest academic Department of Medicine (DOM) in Canada. | Quantitative.  Cross sectional survey. | 41 URM women | Medicine | ***Race/ethnicity-based discrimination:*** social exclusion; higher proportion of URM women felt excluded from informal networks compared with ORM women.  ***Minority tax:*** need to work harder to be respected. Psychological and emotional effects: fear of repercussions; 12.2% URM women vs 23.7% ORM women felt confident that they could address incivility without repercussions. | Structural |
| Pololi (2010) | Race, disadvantage and faculty experiences in academic medicine. | USA | To document URM faculty perceptions and experience of the culture of academic medicine in the US and to raise awareness of obstacles to achieving the goal of having people of color in positions of leadership in academic medicine. | Qualitative. Interviews | Not specified | Medicine | ***Race/ethnicity-based discrimination:*** assumed to be in a lower-level job by both patients and other faculty, low expectations of capability, racism overtly expressed by colleagues.  ***Disparities in representation:*** underrepresentation lead to a sense of invisibility, limited availability of mentors and role models.  ***Minority tax:*** asked to provide service and committee work to promote diversity, need to work hard and prove self to avoid being seen as the token (impact of affirmative action).  ***Psychological and emotional effects:*** isolation, lack of belonging. | Structural |
| Prendergast (2014) | Multiculturalism Policies: Identifying the dialectic of the 'ideal type' within the practices of Canadian nursing. | Canada | To examine the roles of the ideal type and multiculturalism policies within nursing and whether it works in favour of IENs of colour or more as a hindrance to their educational and promotional development. | Qualitative. Interviews.  *THESIS | 10 women nurses of colour | Nursing | ***Race/ethnicity-based discrimination:*** othered (insider vs outsider), accent bias, overscrutinised and overpoliced, devaluation and/or lack of recognition of skills, experiences and credentials, undermining and not given the same level of respect as White colleagues, treated as inferior, occupational segregation; Black nurses placed in menial jobs and White colleagues disproportionately placed in leadership.  ***Minority tax and the ideal type:*** needing to work harder as they were not recognised as the “ideal type”, which some related to being White.  ***Inequitable promotion processes:*** overlooked for promotion, even if qualifications are equivalent to White nurses.  ***Multiculturalism policies:*** multiculturalism beneficial to some in terms of putting nurses of colour into positions. However, multiculturalism also seen as not helping in expanding career opportunities (obscured reality of experiences, could lead to individuals feeling like tokens).  ***Psychological and emotional effects:*** feeling inadequate, lack of belonging, reduced self-confidence, self-blame.  ***Positive supports:*** faith & spirituality, valuing heritage and cultural knowledges, preservation of identities through remembrance of journey to the present.  ***Responses to experiences:*** remaining silent to avoid repercussions, self-advocacy, wanting to be liked by others in order to successfully integrate, renaming and reclaiming the “ideal type” to reflect their own positive attributes. | Structural Representational |
| Prendergast (2024) | Anti-Black racism: Gaining insight into the experiences of Black nurses in Canada. | Canada | To explore how Black nurses experience anti-Black racism while working in Canada’s healthcare system. | Qualitative.  Interviews. | 12 Black women nurses | Nursing | ***Race/ethnicity-based discrimination:*** devalued, qualifications and capabilities questioned, treated differently by White patients, undermined by patients, leadership and staff, patient refusal of care due to nurses’ skin colour, unspoken expectations of low capabilities and lack of confidence, overscrutinised and given harsher punishments for similar mistakes made by White colleagues, organisational passivity to reports of racist experiences.  ***Disparities in representation:*** underrepresentation of Black nurses in places of leadership, inequitable promotion outcomes between White and Black nurses.  ***Invisibility tax:*** rendered invisible, patients do not perceive them to be health professionals.  ***Minority tax:*** need to constantly prove self due to devaluation.  ***Psychological and emotional effects:*** feeling not valued, low sense of belonging, internalisation of racism and adverse perceptions of value and competence. | Structural |
| Price (2005) | The role of cultural diversity climate in recruitment, promotion, and retention of faculty in academic medicine. | USA | To explore the perceptions of physician faculty regarding the following: (1) the institution’s cultural diversity climate and (2) facilitators and barriers to success and professional satisfaction in academic medicine within this context. | Qualitative.  Focus groups, interviews | Total number of minoritised women unknown. Only data explicitly about experiences of minoritised women were extracted | Medicine | ***Race/ethnicity-based discrimination:*** Racialised comments from patients and colleagues, competence questioned, overscrutinised, being "foreign" negatively impacts perceptions of professional competence and promotion opportunities.  ***Gender-based discrimination:*** women assigned more responsibilities than men.  ***Disparities in representation:*** limited number of racial minorities contributes to cycle of underrepresentation; hesitance to enter the profession, and lack of role models to identify with and aspire to.  ***Tokenism:*** racially/ethnically minoritised individuals used to portray an illusion of diversity; however no leadership commitment to supporting the career development of minorities.  ***Responses to experiences:*** need to constantly balance when to confront bias and stereotypes, as this could impact career development. | Structural |
| Ronquillo (2012) | Leaving the Philippines: Oral Histories of Nurses’ Transition to Canadian Nursing Practice. | Canada | To examine the transition experiences of Filipino nurses who immigrated to Canada between 1970 and 2000 | Qualitative. Oral history. Interviews. | 9 Filipino women | Nursing | ***Race/ethnicity-based discrimination:*** overscrutinised due to receiving education outside of Canada. Need to prove self to be worthy of respect. Respect linked to high work ethic. Discrimination by both colleagues and patients. Microaggressions.  ***Cultural ideals:*** centrality of family relationships and family cohesiveness prioritised over career aspiration.  ***Psychological and emotional effects:*** feeling like an outsider, isolation. | Structural |
| Shung-King (2018) | Leadership experiences and practices of South African health managers: what is the influence of gender? -a qualitative, exploratory study | South Africa | To explore the extent to which gender influences the perceptions and experiences of health managers, through a qualitative, exploratory study undertaken in South Africa. | Qualitative.  Case study. Interviews. | 9 Black women | Nursing, Medicine | ***Disparities in representation:*** several women reported being the first Black woman to occupy a leadership position. During journey, women experienced a lack of mentors and role models due to the underrepresentation of women with similar experiences.  ***Disparities in promotion:*** compared to White counterparts, Black women took longer to get into leadership. | Structural |
| Stewart (2009) | Themes of racial discrimination in the experience of Black female nurse managers. | Canada | To explore the question: Are prejudice and discrimination indeed "facts of life" when it comes to the work experiences of Black female nurse managers in Metropolitan Toronto, Canada? Furthermore, how do notions of race influence their everyday experiences? | Qualitative. Interviews  *THESIS | 16 African Canadian women | Nursing | ***Race/ethnicity-based discrimination:*** differential treatment compared to White counterparts, discriminatory behaviours from colleagues, patients, and patient families, work environment perceived as "unfriendly" for REM individuals, competency and abilities questioned and overscrutinised, no credit given for work, needing to work harder than White colleagues, experiences with racism trivialised or dismissed, denied level of respect given to White counterparts; authority and professional status undermined, assumed to be in a lower-level role.  ***Minority tax:*** Need to work harder and over-perform, compared to White counterparts, to prove self and be seen as competent.  ***Psychological and emotional effects:*** stress, feelings of invisibility, burden of fixing the issue of racism, fear of repercussions for challenging racism, fear of confirming stereotypes attributed to Black people, vulnerability.  ***Responses to experiences:*** faith in God/prayer, support networks both within and external to organisation, rationalising experience as something other than racism. | Structural  Representational |
| Sudol (2021) | Prevalence and Nature of Sexist and Racial/Ethnic Microaggressions Against Surgeons and Anesthesiologists. | USA | To examine the prevalence and nature of sexist and racial/ethnic microaggressions against female and racial/ethnic–minority surgeons and anesthesiologists and assess the association with physician burnout. | Quantitative.  Cross sectional survey. | 162 URM women (18 Hispanic, 75 Asian, 17 South Asian, 11 Middle Eastern, 19 Black, 2 Hawaiian/Pacific Islander, 20 Multiracial) | Medicine | ***Race/ethnicity-based discrimination:*** othered; minority women more likely to experience feeling like a foreigner, compared to men. Minority women more likely to experience racial/ethnic sexualisation, compared to men.  ***Microaggressions:*** women experienced a higher prevalence of  microaggressions compared to men. Environmental microaggressions were the most prevalent (i.e. racial underrepresentation, few role models). Microaggressions linked to physician burnout.  ***Psychological and emotional effects:*** feeling like a foreigner/non-belonging, burnout. | Structural |
| Wang (2023) | What's next? On better supporting women of color clinicians in university counseling centers. | USA | To explore the current support they receive, suggestions for University Counselling Centers to better support Women of Color, and suggestions for other Women of Color interested in pursuing a career at University Counselling Centers. | Quantitative.  Survey. | 56 women of colour (9 Asian/Asian American, 25 Black/African American, 12 Hispanic/Latinx, 6 multiracial, 4 Middle Eastern) | Mental Health | ***Organisational support:*** intentional hiring of diverse individuals, opportunities for professional development, ensuring representation of women of colour in leadership roles, acknowledgement and respect of diverse racioethnic identities, leaders and supervisors listen, trust, advocate for, and empower, flexibility to achieve work-life balance and prioritise duties, creation of spaces specifically designated for people of colour.  ***Psychological and emotional effects:*** fear of providing feedback due to potential career consequences. | Structural |

**Table S2: Included non-research sources**

| **Author (Year)** | **Title** | **Country** | **Source Type** | **Healthcare occupations represented** | **Relevant findings** | **Form(s) of intersectionality addressed** |
| --- | --- | --- | --- | --- | --- | --- |
| Ali (2024) | Invisibility and discrimination harm women from ethnic minorities working in the NHS | UK | Opinion | Medicine | ***Racism & discrimination:*** being overlooked/made invisible. Subject to greater scrutiny. Need to work twice as hard for minimal recognition.  ***Microaggressions:*** mistaken for non-medical staff by patients/not assumed to be a doctor. Professionalism and credibility are undermined by stereotypical assumptions and presumptions related to racial/ethnic heritage and cultural/religious practices.  ***Stereotypes:***  presumptions that racially and ethnically minoritised women are oppressed, can’t speak up for themselves, and that religious practices hinder professional duties.  ***Disparities in representation:*** lack of ethnic minority women in positions of leadership. Limited champions, peer support, and networking opportunities.  ***Psychological and emotional effects:*** feelings of inadequacy, invisibility. | Structural  Representational |
| Eke (2021) | Black women in medicine-rising above invisibility. | USA | Perspective | Medicine | ***Limited support:*** under-mentorship.  ***Minority tax:*** asked to lead equity committees and initiatives.  ***Race/ethnicity-based discrimination:*** historically the voices and contributions of Black women health professionals have been dismissed and overlooked. Black women holding medical leadership positions are often automatically assumed to be in a subordinate role.  ***Microaggressions:*** devaluation of achievements, lack of professional recognition. Need to constantly prove self.  ***Psychological and emotional effects:*** chronic stress, frustration, burnout. | Structural |
| Evans (2023) | Two Strikes? A Black Woman’s Experience Working in Healthcare | USA | Web Article | Medicine | ***Disparities in representation:*** underrepresentation in leadership roles results in limited availability of mentors and advocates.  ***Microaggressions:*** expressed verbally or behaviourally.  ***Psychological and emotional effects:*** isolation, distrust, fear, anger, imposter syndrome. | Structural |
| Garrison (2019) | The Lonely Only: Physician Reflections on Race, Bias, and Residency Program Leadership. | USA | Narrative essay | Medicine | ***Race/ethnicity-based discrimination:*** implicit and explicit biases towards racial minorities expressed by colleagues. Friction with colleagues when trying to address racial discrimination. Experiencing a lack of respect and professional regard.  ***Disparities in representation:*** being the sole representative of a racial minority in the workplace and in leadership.  ***Psychological and emotional effects*:** isolation, anger, frustration, and shame. | Structural |
| Gooden (2017) | As a black woman you have to work twice as hard to get to where you want to be'. | UK | Opinion | Nursing/midwifery | ***Race/ethnicity-based discrimination:*** ideas and suggestions dismissed unless presented by a man.  ***Stereotypes:*** Black women viewed as less competent and lazy.  ***Tokenism:*** Black midwives used to boost numbers within the organisation and treated badly once within.  ***Minority tax:*** perception of needing to work twice as hard to achieve the same things as White counterparts. ***Psychological and emotional effects*:** internalisation of discriminatory perceptions. | Structural  Representational |
| Jung (2005) | The life of a Chinese American female physician executive | USA | Article | Medicine | ***Gender-based discrimination*:** sexism; suggestive comments from male surgeons, negative views about the choice to have children.  ***Lack of support:*** limited access to role models, career pathway previously uncharted. | Structural |
| Lacorte (2021) | Colleague Conversations: Women & Leadership in Hematology/Oncology. | USA | Article | Medicine | ***Race/ethnicity-based discrimination:*** need to frequently justify credentials and professional decisions. Assumed to be in a lower-level/service job due to ethnicity.  ***Minority tax:*** need to work twice as hard to prove competence. | Structural |
| O'Dowd (2022) | Challenging prejudice: the chief medical officer and consultant psychiatrist | UK | Article | Medicine | ***Race/ethnicity-based discrimination:*** discriminated against due to being an immigrant.  ***Limited organisational support:*** limited number of allies to provide support and advocacy. | Structural |
| Thomas (2019) | A Dark Spot on a White Canvas: Thoughts on Being a Black Academic Psychiatrist | USA | Perspective | Medicine | ***Disparities in representation:*** being the only visible minority at different points in career journey.  ***Invisibility paradox:*** heightened visibility but this is countered by the feeling/reality of voice not being relevant or considered.  ***Tokenism:*** feelings of only being present at meetings to "add some colour to the room”, voice and contributions dismissed. Voice is often silenced.  ***Minority tax:*** being asked to address diversity issues. Perceived obligation to alleviate the guilt of White colleagues.  ***Psychological and emotional effects*:** isolation, anger, resentment, impostor syndrome, sense of not belonging, low sense of worth to organisation, self-silencing. | Structural |
